# Lunar chronology model with the Chang’e-6 farside samples and implications for the early impact history

**DOI:** 10.1126/sciadv.ady9265

**Published:** 2026-02-04

**Authors:** Zongyu Yue, Sheng Gou, Yexin Wang, Huacheng Li, Gregory Michael, Jianzhong Liu, Shujuan Sun, Yangting Lin, Kaichang Di, Qiuli Li, Yi Chen, Wei Yang, Bin Xie, Sen Hu, Shouding Li, Bo Zheng, Tianqiao Mao, Xianhua Li, Fuyuan Wu

**Affiliations:** ^1^ Key Laboratory of Planetary Science and Frontier Technology, Institute of Geology and Geophysics, Chinese Academy of Sciences, Beijing 100029, China.; ^2^ State Key Laboratory of Remote Sensing and Digital Earth, Aerospace Information Research Institute, Chinese Academy of Sciences, Beijing 100101, China.; ^3^ Institute of Geochemistry, Chinese Academy of Sciences, Guiyang 550081, China.; ^4^ School of Architecture and Civil Engineering, Chengdu University, Chengdu 610106, China.; ^5^ State Key Laboratory of Lithospheric and Environmental Coevolution, Institute of Geology and Geophysics, Chinese Academy of Sciences, Beijing 100029, China.; ^6^ Key Laboratory of Deep Petroleum Intelligent Exploration and Development, Institute of Geology and Geophysics, Chinese Academy of Sciences, Beijing 100029, China.

## Abstract

The radiometric ages of the returned samples are the cornerstone of lunar cratering chronology models. However, all the previous samples were from the lunar nearside and the radiometric ages of those samples that can be associated with particular surfaces are <4.0 billion years. On 25 June 2024, Chang’e-6 successfully returned 1.935-kilogram samples from the lunar farside. The samples included local basalts with an age of 2807 ± 3 million years and the norites with an age of 4247 ± 5 million years likely corresponding to the age of the South Pole–Aitken basin. With these radiometric ages, we refined the lunar chronology function (CF) and verified that it is still consistent with a combination of an exponential decrease and a linear rate. We further derived the impacting rate and found it supports a smooth decay instead of abrupt changes of the impactor flux at early times. The refined lunar CF can be used to obtain more reliable ages for unsampled lunar areas and provide critical constraint for the lunar early impact history.

## Teaser

Ages of Chang’e-6 farside samples refine lunar cratering chronology model and indicate a smooth decline in early impact rate.

## INTRODUCTION

Absolute ages are crucial for understanding the evolution of solar system bodies, but direct radiometric dating has thus far been extensively applied only to Earth. For unsampled regions on the Moon, absolute model ages (AMAs) are usually estimated by the crater size–frequency distribution (CSFD) method, e.g., refs. (*1*–*4*). As the core component of the CSFD method, the lunar chronology model [i.e., chronology function (CF)] depicts the relationship between the radiometric ages of the returned samples and the corresponding crater distribution densities at the sampling sites, e.g., refs. (*1*, *5*–*7*). The cornerstone of lunar chronology model is the measured radiometric ages of the returned lunar samples. However, the samples used in the current lunar CF are all from the Moon’s nearside (*1*, *8*). Samples from the lunar farside are essential to validate the CF’s applicability across the entire Moon. Moreover, the oldest samples currently used to establish the lunar CF are subject to considerable controversy (Supplementary Note 1). Thus, there is an urgent need for samples from the lunar farside and old geologic unit to determine whether the current model is applicable throughout the Moon and accurately represents the earlier lunar impacts.

As the first sample return mission in the human history from the lunar farside, China’s Chang’e-6 (CE-6) was launched on 3 May 2024, and it landed in the Apollo basin within the South Pole–Aitken (SPA) basin on the Moon on 2 June 2024 (figs. S1 and S2). The SPA basin is the largest and oldest impact basin on the Moon, and numerical simulations of the formation of the SPA basin suggest that extensive molten mantle material emerged and was redistributed across the impact basin floor (*9*, *10*). The subsequent differentiation process likely resulted in an upper layer of noritic composition (*11*), aligning with spectral analyses of materials ranging from noritic (*12*) to mafic (*13*). The landing site (153.9856°W, 41.6383°S) is located in the southern basalt unit within the Apollo basin (fig. S1). Previous remote sensing studies have divided this basalt area into different units according to the mineral compositions (*14*–*17*), and CE-6 landed on the west part of the basalt unit (fig. S2). After landing on the Moon, CE-6 began scooping surface samples using a robotic arm and drilling subsurface samples. As a result, 1935.3-g samples were collected and were successfully returned to Earth on 25 June 2024 (*18*).

## RESULTS

### Refined lunar chronology model

We have conducted mineral and radiometric dating analyses of the scooped surface samples immediately after they were allocated by the China National Space Administration. Radiometric measurements using the Pb-Pb dating technique were made on both Zr-rich minerals and phosphates of the basalt samples and revealed the absolute age of the basalt unit where CE-6 landed was 2807 ± 3 million years (Ma) (*19*). This result is consistent to that reported in ref. (*20*). In addition, the norite samples plausibly represent the differentiated products of the SPA impact melt pool and they have been radiometrically dated to be 4247 ± 5 Ma (*21*). The corresponding crater frequencies are described in detail in Supplementary Note 1. It is worth noting that, when calculating the crater frequency for SPA basin, basaltic regions within the SPA basin should be excluded because their ages are notably younger than the SPA impact event. Larger craters should be preferentially used to minimize the influence of secondary craters, and a buffered non–sparseness correction method should be applied to account for the erosion of earlier craters by later-formed ones. These radiometric ages and their corresponding crater frequencies were then used to update the currently widely used lunar CF with the crater densities of the corresponding geological units.

Neukum (*1*) first fitted the lunar CF between the radiometric ages of Apollo and Luna samples and the crater frequencies *N*(1) in the corresponding areas, i.e., the total number of craters with diameter ≥ 1 km/km^2^. However, more modern calibration points were derived with recently acquired high-resolution images, e.g., refs. (*8*, *22*–*25*), in which both the sample ages and the corresponding *N*(1) values are updated. We carefully reviewed the relevant progresses and selected the most reliable results for use in this study (Supplementary Note 1). Table 1 lists the calibration points used by Neukum (*1*) and this research.

**Table 1.** Calibration points used in the Neukum model and this research. The references for the calibration points are explained in Supplementary Note 1.

Site and mission	Chronology calibrations		Chronology calibrations in this study	
	*N*(1) (km^−2^)	Age (Ga)	*N*(1) (km^−2^)	Age (Ga)
Highland (Terrae)	(3.6 ± 1.1) × 10^−1^	4.35 ± 0.10		
Nectaris Basin (A16)	(1.2 ± 0.4) × 10^−1^	4.10 ± 0.10		
Apennines (A15)	(3.5 ± 0.5) × 10^−2^	3.91 ± 0.10		
Descartes Formation (A16)	(3.4 ± 0.7) × 10^−2^	3.90 ± 0.10		
Fra Mauro Formation (A14)	(3.7 ± 0.7) × 10^−2^	3.91 ± 0.10	(3.82 ± 1.07) × 10^−2^	3.922 ± 0.012 (U-Pb dating)
Taurus Littrow Mare (A17)	(1.0 ± 0.3) × 10^−2^	3.70 ± 0.10	(1.06 ± 0.21) × 10^−2^	3.752 ± 0.007 (Pb-Pb dating)
Mare Tranquillitatis (old) (A11)	(9.0 ± 1.8) × 10^−3^	3.72 ± 0.10		
Mare Tranquillitatis (young) (A11)	(6.4 ± 2.0) × 10^−3^	3.53 ± 0.05	(6.64 ± 0.561) × 10^−3^	3.578 ± 0.009 (Pb-Pb dating)
Mare Imbrium (A15)	(3.2 ± 1.1) × 10^−3^	3.28 ± 0.10	(2.23 ± 0.12) × 10^−3^	3.281 ± 0.012 (Pb-Pb dating)
Oceanus Procellarum (A12)	(3.6 ± 1.1) × 10^−3^	3.18 ± 0.10	(2.34 ± 0.05) × 10^−3^	3.242 ± 0.013 (Pb-Pb dating)
Mare Fecunditatis (L16)	(3.3 ± 1.0) × 10^−3^	3.40 ± 0.04	(4.32 ± 0.01) × 10^−3^	3.382 ± 0.014 (Ar-Ar dating)
Mare Crisium (L24)	(3.0 ± 0.6) × 10^−3^	3.30 ± 0.10	(2.54 ± 0.08) × 10^−3^	3.328 ± 0.021 (Ar-Ar dating)
Copernicus (A12)	(1.3 ± 0.3) × 10^−3^	0.85 ± 0.2	(6.68 ± 0.048) × 10^−>4^	0.80 ± 0.015 (Ar-Ar dating)
Tycho (A17)	(9.0 ± 1.8) × 10^−5^	0.109 ± 0.004	(7.12 ± 0.063) × 10^−5^	0.109 ± 0.004 (Kr-Kr dating)
North Ray (A16)	(4.4 ± 1.1) × 10^−5^	0.0500 ± 0.0014	(3.90 ± 0.043) × 10^−5^	0.0503 ± 0.0008 (Kr-Kr dating)
Cone (A14)	(2.1 ± 0.5) × 10^−5^	0.0260 ± 0.0008	(2.1 ± 0.5) × 10^−5^	0.0260 ± 0.0008 (Kr-Kr dating)
Phanerozoic craters (North America + Europe, lunar equivalent)	(3.6 ± 1.1) × 10^−4^	0.375 ± 0.075		
Northern Oceanus Procellarum (CE-5)			(1.74 ± 0.022) × 10^−3^	2.030 ± 0.004 (Pb-Pb dating)
Mare Apollo (CE6)			(2.08 ± 0.13) × 10^−3^	2.807 ± 0.003 (Pb-Pb dating)
SPA basin (CE6)			(3.69 ± 0.48) × 10^−1^	4.247 ± 0.005 (Pb-Pb dating)

Figure S2 shows the two radiometric ages and corresponding *N*(1) values from the farside are within 95% confidence region of the lunar CF that were derived from the nearside data (Table 1). This suggests that the farside impact flux is similar to that of the nearside, which is consistent with previous theoretical analyses, e.g., refs. (*6*, *26*, *27*). The CE-6 samples supported the impact symmetry between the lunar nearside and farside, which provides a foundation to establish a universal crater CF cross the whole Moon. Therefore, the two radiometric ages and corresponding *N*(1) values along with previous datasets from Apollo, Luna, and Chang’e-5 missions (Table 1) are used to update the lunar CF through least-squares fitting. The resultant lunar cratering CF is as follows

$$N\left(1,t\right)=3.885\times 10^{-15}\left(e^{7.595t}-1\right)+7.377\times 10^{-4}t$$ (1)

**Fig. 1.**
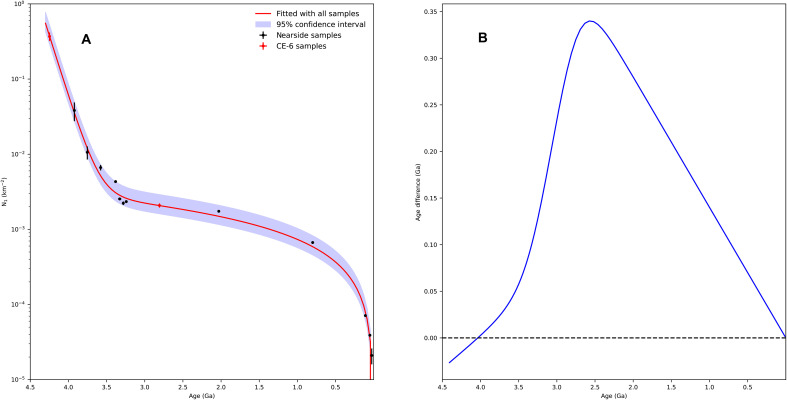
Refined lunar chronology model and comparison with a previous model. (**A**) Refined lunar chronology model and 95% confidence interval along with all the radiometric ages. (**B**) Model age difference of the refined model with respect to the Neukum (*1*) model.

Figure 1A shows the fitted model with all the data points. Figure 1B illustrates the model age differences between the refined model and the most widely used Neukum (*1*) model (i.e., refined model–Neukum model) with respect to the age derived from the Neukum (*1*) model. Compared with the Neukum (*1*) model, the refined model with the CE-6 samples gives older ages in most of the geologic ages regarding to the same crater frequency, whereas the case is reverse when the ages are >4.05 billion years (Ga). The maximum difference is 0.34 Ga at 2.58 Ga, which is well within the uncertainties of crater chronologies. It is noteworthy to observe that all of the datasets are within the 95% confidence interval of the refined lunar CF, indicating the good performance of the fitting. In addition, we also used the lunar meteorite age of 4.33 Ga (*28*) as the SPA basin age to fit the lunar CF, and there is not too much difference between them (the maximum difference is about 0.09 Ga; fig. S6).

From the refined lunar cratering chronology model (*1*), the derived impact rate for *D* ≥ 1 km is as follows

$$\phi \left(1,t\right)=2.951\times 10^{-14}e^{7.595t}+7.377\times 10^{-4}$$ (2)

**Fig. 2.**
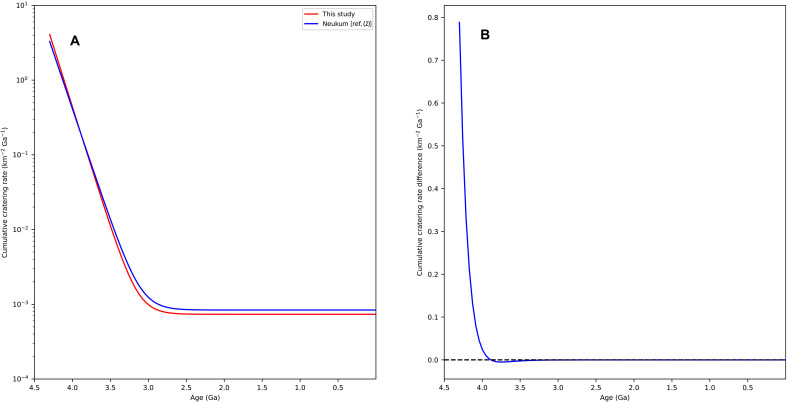
Cratering rate and the difference between the refined model and the Neukum model. (**A**) Cumulative cratering rates with respect to the model age of the refined model (red) and Neukum (*1*) model (blue). (**B**) Difference of the cumulative cratering rates between the two models [refined model–Neukum (*1*) model].

The result is shown in Fig. 2A in comparison with the model by Neukum (*1*). The frequency of impact events that produce craters 1 km or larger likewise exhibits an exponential decline before ~3.0 Ga and attains an almost invariant value afterward. The disparity in the impact rates yielding craters 1 km and larger between the two models is clearly illustrated in Fig. 2B. Before 3.91 Ga, the cumulative cratering rate derived from the refined model consistently surpasses that of the Neukum (*1*) model, with the divergence intensifying toward earlier epochs. Since 3.91 Ga to the present, the refined model presents a slightly lower cumulative impact rate and the maximum difference appears at 3.78 Ga. However, from 3.78 Ga to the present, the difference between the two models quickly diminishes until they are almost identical.

## DISCUSSION

### Implication for lunar early impact history

**Fig. 3.**
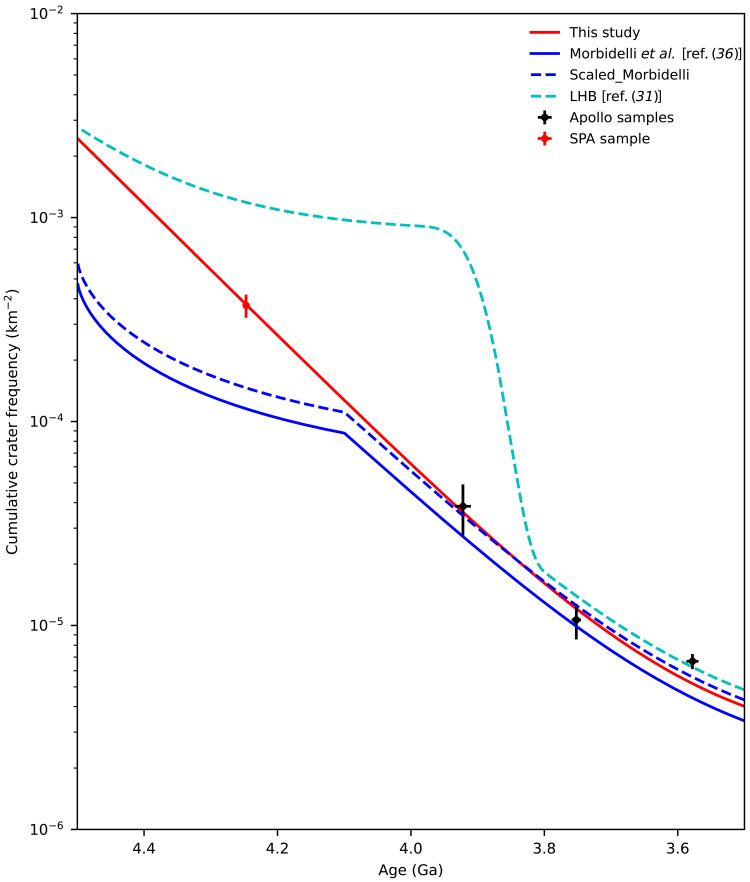
Comparison of the cumulative crater frequency *N*(>20 km) between previous models and the result in this study. The red solid line represents the result obtained using Eq. 1 in this paper, as well as the relationship between *N*(20) and *N*(1); the blue solid line is the result from ref. (*36*), and the blue dashed line represents the result normalized to this study based on the cumulative impact rate between 3.5 and 4.1 Ga; the cyan dashed line shows the results for the LHB from ref. (*31*). The black dots indicate measurements from Apollo samples, whereas the red cross represents the result from the potential SPA sample returned by the CE-6 mission.

The oldest sample from CE-6, assumed to represent the SPA impact event (*21*), was used in fitting the refined CF model, providing a unique opportunity to constrain the early lunar impact history. Now, there are several hypotheses on the early impact history of the Moon, including the monotonically decreasing impact flux (*1*, *29*–*31*), the Late Heavy Bombardment (LHB) at about 3.9 Ga (*32*–*35*), a sawtooth-like uptick impact flux earlier than about 4.1 Ga (*36*), etc. The hot debate on the early impact history of the Moon has further extended to the dispute over the source of lunar impactors, e.g., refs. (*37*–*39*). The reason for this controversy is that there were very few samples dating impacts before 4.0 Ga ago and a widespread signature of isotopic disturbance in the samples of ~3.9 Ga ago (*39*). CE-6 has arguably returned samples from the SPA basin, plausibly providing an important “anchor point” to address this issue. Figure 3 shows the comparison between the crater CF calibrated with the proposed SPA age and others (Table 1). The blue solid line is the sawtooth-like model (*36*), and the blue dashed line is translated to the refined model according to the cumulative cratering rate between 3.5 and 4.1 Ga (see Materials and Methods). It can be observed that the SPA age obviously deviates from the sawtooth-like model. Substantial deviations also appear from the LHB model, and similar situations also appear in the samples of the Fra Mauro Formation (3.922 Ga). Therefore, the new observations do not support these theoretical models with abrupt changes. If we adopt the 4.33 Ga age of the lunar meteorite for the SPA basin (*28*), the result also does not support the LHB model (fig. S7).

In summary, a refined lunar cratering chronology model based on the radiometric ages of CE-6 samples is presented, assuming one of them represents the time of the SPA impact. The result indicates that a summation of an exponential equation and a linear equation is applicable to describe the chronology model, and correspondingly the impact rate rapidly decreased before about 3.0 Ga and then gradually slowed down to its current level. No clear evidence from the newly returned samples was found to support that the impact flux on the Moon sharply increased around 3.9 Ga ago. Overall, the refined lunar chronology model is not substantially different from the widely used Neukum (*1*) model, which was based solely on samples from the nearside of the Moon. This suggests that the impact flux on the lunar nearside does not differ substantially to that on the farside. The refined model will enhance the reliability when estimating ages with the CSFD method, and we suggest that they be used in lunar surface dating in the future.

## MATERIALS AND METHODS

### Lunar crater CF fitting

Like the previous lunar crater CF proposed by Neukum (*1*), the current model is also expressed as the sum of an exponential equation and a linear equation. The coefficients of the CF were solved iteratively using the nonlinear least-squares fitting algorithm (*40*, *41*), with the coefficients of the Neukum (*1*) model being the initial values. The residual function used in the nonlinear fitting is expressed as follows

$$\mathrm{err}=1-\frac{f\left(x_{i}\right)}{y_{i}}$$ (3)

where $f\left(x_{i}\right)$ represents the *i*th *N*(1) value obtained from the fitting function, and $y_{i}$ is the *i*th observed *N*(1) value. The errors obtained from the above function follow a normal distribution according to the Shapiro-Wilk test (*42*), indicating that the above nonlinear fitting algorithm has good performance. The fitting result is shown in Fig. 1, and the comparison of the fitting result with other models can be seen in figs. S3 and S4.

To analyze whether the datasets related with the two samples on the lunar farside is consistent with the lunar CF only with the lunar nearside samples, we first calculated the 95% confidence interval of the fitted lunar CF and it can be expressed as follows

$$\frac{f\left(x_{i}\right)}{1+1.96\sigma }<y<\frac{f\left(x_{i}\right)}{1-1.96\sigma }$$ (4)

where σ is the SE during the fitting, and $f\left(x_{i}\right)$ is the calculated *N*(1) value according to the fitting function. The above result, along with the datasets from CE-6 samples, is shown in fig. S1. The CE-6 data points fall within the 95% confidence interval of the lunar crater CF curve obtained using only samples from the lunar nearside, indicating a high degree of consistency between the impact flux on the farside of the Moon and that on the nearside.

### Translation of the sawtooth-like model

There is a systematic offset between the sawtooth-like model provided in ref. (*36*) and the refined model obtained in this paper. To facilitate comparison of the impact flux predictions for the early Moon, it would be best to translate the sawtooth-like model so that the curves align roughly within the time period less than 4.1 Ga. The specific method to achieve this translation is based on the systematic offset between the two models since 4.1 Ga. The approach is to find an optimal factor that, when multiplied by the entire curve, achieves the translation.

Generally, if two CFs [denoted as $N_{s}\left(t\right)$ and $N_{n}\left(t\right)$] overlap at only one age $t_{1}$, the $N_{n}\left(t\right)$ CF can be translated to $N_{s}\left(t\right)$ CF by simply multiplying a scaling factor $c=\frac{N_{s}\left(t_{1}\right)}{N_{n}\left(t_{1}\right)}$. The CFs of $N_{s}\left(t\right)$ and $N_{n}\left(t\right)$ overlap over many ages, and the goal is to find the optimal factor so that the two CF curves coincide as closely as possible after translation. In this study, the sawtooth-like model and the refined model are considered to be consistent since 4.1 Ga. Therefore, the optimal factor $\bar{c}$ should satisfy the following condition: After translating the sawtooth-like model, the sum of the squared differences between the two CFs at the same ages should be minimized. This is the residual function, based on which the optimal factor is expressed as follows

$$\bar{c}=\frac{\sum_{i=1}^{m}\left[N_{s}\left(t_{i}\right)\cdot N_{n}\left(t_{i}\right)\right]}{\sum_{i=1}^{m}{\left[N_{n}\left(t_{i}\right)\right]}^{2}}$$ (5)

where *m* represents the total number of ages used for the translation.

## Supplementary Materials

### The PDF file includes:

Supplementary Note 1 Figs. S1 to S7 Legend for code S1 References

### Other Supplementary Material for this manuscript includes the following:

Code S1

## References

[1] G. Neukum, “Meteoriten bombardement und Datierung planetarer Oberflächen (Meteorite bombardment and dating of planetary surfaces),” thesis, Ludwig-Maximilians University, Munich, Germany (1983).

[2] H. Hiesinger, J. W. Head III, U. Wolf, R. Jaumann, G. Neukum, Ages and stratigraphy of lunar mare basalts in Mare Frigoris and other nearside maria based on crater size-frequency distribution measurements. J. Geophys. Res. Planets 115, E03003 (2010).

[3] J. Whitten, J. W. Head, M. Staid, C. M. Pieters, J. Mustard, R. Clark, J. Nettles, R. L. Klima, L. Taylor, Lunar mare deposits associated with the Orientale impact basin: New insights into mineralogy, history, mode of emplacement, and relation to Orientale Basin evolution from Moon Mineralogy Mapper (M^3^) data from Chandrayaan-1. J. Geophys. Res. Planets 116, E00G09 (2011).

[4] M. R. Kirchoff, C. R. Chapman, S. Marchi, K. M. Curtis, B. Enke, W. F. Bottke, Ages of large lunar impact craters and implications for bombardment during the Moon’s middle age. Icarus 225, 325–341 (2013).

[5] R. Wagner, J. W. Head III, U. Wolf, G. Neukum, Stratigraphic sequence and ages of volcanic units in the Gruithuisen region of the Moon. J. Geophys. Res. Planets 107, 14-1–14-15 (2002).

[6] M. Le Feuvre, M. A. Wieczorek, Nonuniform cratering of the Moon and a revised crater chronology of the inner Solar System. Icarus 214, 1–20 (2011).

[7] W. K. Hartmann, R. Strom, S. Weidenschilling, K. Blasius, K. Jones, in *Basaltic Volcanism on the Terrestrial Planets (Basaltic Volcanism Study Project)* (Pergamon Press, 1981), pp. 1050–1127.

[8] D. Stöffler, G. Ryder, Stratigraphy and isotope ages of lunar geologic units: Chronological standard for the inner solar system. Space Sci. Rev. 96, 9–54 (2001).

[9] R. W. K. Potter, G. S. Collins, W. S. Kiefer, P. J. McGovern, D. A. Kring, Constraining the size of the South Pole-Aitken basin impact. Icarus 220, 730–743 (2012).

[10] R. I. Citron, D. E. Smith, S. T. Stewart, L. L. Hood, M. T. Zuber, The South Pole-Aitken basin: Constraints on impact excavation, melt, and ejecta. Geophys. Res. Lett. 51, e2024GL110034 (2024).

[11] W. M. Vaughan, J. W. Head, Impact melt differentiation in the South Pole-Aitken basin: Some observations and speculations. Planet. Space Sci. 91, 101–106 (2014).

[12] C. M. Pieters, J. W. Head, L. Gaddis, B. Jolliff, M. Duke, Rock types of South Pole-Aitken basin and extent of basaltic volcanism. J. Geophys. Res. Planets 106, 28001–28022 (2001).

[13] B. R. Hawke, C. A. Peterson, D. T. Blewett, D. B. J. Bussey, P. G. Lucey, G. J. Taylor, P. D. Spudis, Distribution and modes of occurrence of lunar anorthosite. J. Geophys. Res. Planets 108, 5050 (2003).

[14] J. H. Pasckert, H. Hiesinger, C. H. van der Bogert, Lunar farside volcanism in and around the South Pole–Aitken basin. Icarus 299, 538–562 (2018).

[15] X. Zeng, D. Liu, Y. Chen, Q. Zhou, X. Ren, Z. Zhang, W. Yan, W. Chen, Q. Wang, X. Deng, H. Hu, J. Liu, W. Zuo, J. W. Head, C. Li, Landing site of the Chang’e-6 lunar farside sample return mission from the Apollo basin. Nat. Astron. 7, 1188–1197 (2023).

[16] Y. Qian, J. Head, J. Michalski, X. Wang, C. H. van der Bogert, H. Hiesinger, L. Sun, W. Yang, L. Xiao, X. Li, G. Zhao, Long-lasting farside volcanism in the Apollo basin: Chang’e-6 landing site. Earth Planet. Sci. Lett. 637, 118737 (2024).

[17] Y. Wang, J. Nan, C. Zhao, B. Xie, S. Gou, Z. Yue, K. Di, H. Zhang, X. Deng, S. Sun, A catalogue of impact craters and surface age analysis in the Chang’e-6 landing area. Remote Sens. 16, 2014 (2024).

[18] C. Li, H. Hu, M.-F. Yang, J. Liu, Q. Zhou, X. Ren, B. Liu, D. Liu, X. Zeng, W. Zuo, G. Zhang, H. Zhang, S. Yang, Q. Wang, X. Deng, X. Gao, Y. Su, W. Wen, Z. Ouyang, Nature of the lunar farside samples returned by the Chang’E-6 mission. Natl. Sci. Rev. 11, nwae328 (2024). 39440270 10.1093/nsr/nwae328 PMC11495410

[19] Q. W. L. Zhang, M.-H. Yang, Q.-L. Li, Y. Liu, Z.-Y. Yue, Q. Zhou, L.-Y. Chen, H.-X. Ma, S.-H. Yang, X. Tang, G.-L. Zhang, X. Ren, X.-H. Li, Lunar farside volcanism 2.8 billion years ago from Chang’e-6 basalts. Nature 643, 356–360 (2025). 39547260 10.1038/s41586-024-08382-0 PMC12240805

[20] Z. Cui, Q. Yang, Y.-Q. Zhang, C. Wang, H. Xian, Z. Chen, Z. Xiao, Y. Qian, J. W. Head, C. R. Neal, L. Xiao, F. Luo, J. Chen, P. He, Y. Cao, Q. Zhou, F. Huang, L. Chen, B. Wei, J. Wang, Y.-N. Yang, S. Li, Y. Yang, X. Lin, J. Zhu, L. Zhang, Y.-G. Xu, A sample of the Moon’s far side retrieved by Chang’e-6 contains 2.83-billion-year-old basalt. Science 386, 1395–1399 (2024). 39545330 10.1126/science.adt1093

[21] B. Su, Y. Chen, Z. Wang, D. Zhang, H. Chen, S. Gou, Z. Yue, Y. Liu, J. Yuan, G.-Q. Tang, S. Guo, Q. Li, Y.-T. Lin, X.-H. Li, F.-Y. Wu, South Pole–Aitken massive impact 4.25 billion years ago revealed by Chang’e-6. Natl. Sci. Rev. 12, nwaf103 (2025). 40391146 10.1093/nsr/nwaf103 PMC12086667

[22] S. Marchi, S. Mottola, G. Cremonese, M. Massironi, E. Martellato, A new chronology for the Moon and Mercury. Astron. J. 137, 4936–4948 (2009).

[23] S. J. Robbins, New crater calibrations for the lunar crater-age chronology. Earth Planet. Sci. Lett. 403, 188–198 (2014).

[24] H. Hiesinger, C. H. van der Bogert, J. H. Pasckert, L. Funcke, L. Giacomini, L. R. Ostrach, M. S. Robinson, How old are young lunar craters? J. Geophys. Res. Planets 117, E00H10 (2012).

[25] S. C. Werner, B. Bultel, T. Rolf, Review and revision of the lunar cratering chronology—Lunar timescale part 2. Planet. Sci. 4, 147 (2023).

[26] L. W. Bandermann, S. F. Singer, Calculation of meteoroid impacts on moon and earth. Icarus 19, 108–113 (1973).

[27] H. Li, N. Zhang, Z. Yue, Y. Zhang, Lunar cratering asymmetries with high lunar orbital obliquity and inclination of the Moon. Res. Astron. Astrophys. 21, 140 (2021).

[28] K. H. Joy, N. Wang, J. F. Snape, A. Goodwin, J. F. Pernet-Fisher, M. J. Whitehouse, Y. Liu, Y. T. Lin, J. R. Darling, P. Tar, R. Tartèse, Evidence of a 4.33 billion year age for the Moon’s South Pole–Aitken basin. Nat. Astron. 9, 55–65 (2024). 39866548 10.1038/s41550-024-02380-y PMC11757148

[29] G. Neukum, B. A. Ivanov, W. K. Hartmann, Cratering records in the inner solar system in relation to the lunar reference system. Space Sci. Rev. 96, 55–86 (2001).

[30] W. K. Hartmann, Megaregolith evolution and cratering cataclysm models—Lunar cataclysm as a misconception (28 years later). Meteorit. Planet. Sci. 38, 579–593 (2003).

[31] G. Michael, A. Basilevsky, G. Neukum, On the history of the early meteoritic bombardment of the Moon: Was there a terminal lunar cataclysm? Icarus 302, 80–103 (2018).

[32] F. Tera, D. A. Papanastassiou, G. J. Wasserburg, Isotopic evidence for a terminal lunar cataclysm. Earth Planet. Sci. Lett. 22, 1–21 (1974).

[33] B. A. Cohen, T. D. Swindle, D. A. Kring, Support for the lunar cataclysm hypothesis from lunar meteorite impact melt ages. Science 290, 1754–1756 (2000). 11099411 10.1126/science.290.5497.1754

[34] S. Marchi, W. F. Bottke, D. A. Kring, A. Morbidelli, The onset of the lunar cataclysm as recorded in its ancient crater populations. Earth Planet. Sci. Lett. 325-326, 27–38 (2012).

[35] M. D. Norman, A. A. Nemchin, A 4.2 billion year old impact basin on the Moon: U-Pb dating of zirconolite and apatite in lunar melt rock 67955. Earth Planet. Sci. Lett. 388, 387–398 (2014).

[36] A. Morbidelli, S. Marchi, W. F. Bottke, D. A. Kring, A sawtooth-like timeline for the first billion years of lunar bombardment. Earth Planet. Sci. Lett. 355-356, 144–151 (2012).

[37] B. A. Ivanov, Mars/Moon cratering rate ratio estimates. Space Sci. Rev. 96, 87–104 (2001).

[38] W. F. Bottke, D. Vokrouhlický, D. Minton, D. Nesvorný, A. Morbidelli, R. Brasser, B. Simonson, H. F. Levison, An Archaean heavy bombardment from a destabilized extension of the asteroid belt. Nature 485, 78–81 (2012). 22535245 10.1038/nature10967

[39] C. I. Fassett, D. A. Minton, Impact bombardment of the terrestrial planets and the early history of the Solar System. Nat. Geosci. 6, 520–524 (2013).

[40] T. F. Coleman, Y. Li, On the convergence of interior-reflective Newton methods for nonlinear minimization subject to bounds. Math. Program. 67, 189–224 (1994).

[41] T. F. Coleman, Y. Li, An interior trust region approach for nonlinear minimization subject to bounds. SIAM J. Optim. 6, 418–445 (1996).

[42] S. S. Shapiro, M. B. Wilk, An analysis of variance test for normality (complete samples). Biometrika 52, 591–611 (1965).

[43] A. Lagain, H. A. R. Devillepoix, P. Vernazza, D. Robertson, M. Granvik, P. Pokorny, A. Ozerov, P. M. Shober, L. Jorda, K. Servis, J. H. Fairweather, Y. Quesnel, G. K. Benedix, Recalibration of the lunar chronology due to spatial cratering-rate variability. Icarus 411, 115956 (2024).

[44] M. D. Norman, The lunar cataclysm: Reality or “mythconception”? Elements 5, 23–28 (2009).

[45] J. W. Head, Stratigraphy of the Descartes region (Apollo 16): Implications for the origin of samples. The Moon 11, 77–99 (1974).

[46] G. Neukum, B. A. Ivanov, in *Hazards Due to Comets and Asteroids*, T. Gehrels, M. S. Matthews, A. M. Schumann, Eds. (Univ. of Arizona Press, 1994), pp. 359–416.

[47] D. Stöffler, A. Bischoff, R. Borchardt, A. Burghele, A. Deutsch, E. K. Jessberger, R. Ostertag, H. Palme, B. Spettel, W. U. Reimold, K. Wacker, H. Wänke, Composition and evolution of the lunar crust in the Descartes Highlands, Apollo 16. J. Geophys. Res. Solid Earth 90, C449–C506 (1985).

[48] D. E. Wilhelms, J. F. McCauley, N. J. Trask, *The Geologic History of the Moon* (US Government Printing Office, 1987).

[49] G. Dalrymple, G. Ryder, R. Duncan, J. Huard, “^40^Ar-^39^Ar ages of Apollo 16 impact melt rocks by laser step heating,” in the *32nd Lunar and Planetary Science Conference* (Lunar and Planetary Institute, 2001).

[50] G. Ryder, Mass flux in the ancient Earth-Moon system and benign implications for the origin of life on Earth. J. Geophys. Res. Planets 107, 6-1–6-13 (2002).

[51] P. D. Spudis, Apollo 16 site geology and impact melts: Implications for the geologic history of the lunar highlands. J. Geophys. Res. Solid Earth 89, C95–C107 (1984).

[52] A. A. Nemchin, T. Long, B. L. Jolliff, Y. Wan, J. F. Snape, R. Zeigler, M. L. Grange, D. Liu, M. J. Whitehouse, N. E. Timms, F. Jourdan, Ages of lunar impact breccias: Limits for timing of the Imbrium impact. Geochemistry 81, 125683 (2021).

[53] A. A. Nemchin, R. T. Pidgeon, D. Healy, M. L. Grange, M. J. Whitehouse, J. Vaughan, The comparative behavior of apatite-zircon U-Pb systems in Apollo 14 breccias: Implications for the thermal history of the Fra Mauro Formation. Meteorit. Planet. Sci. 44, 1717–1734 (2009).

[54] R. E. Merle, A. A. Nemchin, M. L. Grange, M. J. Whitehouse, R. T. Pidgeon, High resolution U-Pb ages of Ca-phosphates in Apollo 14 breccias: Implications for the age of the Imbrium impact. Meteorit. Planet. Sci. 49, 2241–2251 (2014).

[55] S. C. Werner, B. Bultel, T. Rolf, V. Assis Fernandes, Orientale ejecta at the Apollo 14 landing site implies a 200-million-year stratigraphic time shift on the Moon. Planet. Sci. 3, 65 (2022).

[56] W. Iqbal, H. Hiesinger, D. Borisov, C. H. van der Bogert, J. W. Head, Geological mapping and chronology of lunar landing sites: Apollo 14. Icarus 406, 115732 (2023).

[57] J. M. Rhodes, N. J. Hubbard, H. Wiesmann, K. V. Rodgers, J. C. Brannon, B. M. Bansal, “Chemistry, classification, and petrogenesis of Apollo 17 mare basalts,” in the *7th Lunar Science Conference* (Lunar and Planetary Institute/Pergamon Press, 1976).

[58] W. M. Kaula, J. W. Head III, R. B. Merrill, R. O. Pepin, S. C. Solomon, D. Walker, C. A. Wood, *Basaltic Volcanism on the Terrestrial Planets* (Pergamon Press, 1981), 1286 pp.

[59] J. F. Snape, A. A. Nemchin, M. J. Whitehouse, R. E. Merle, T. Hopkinson, M. Anand, The timing of basaltic volcanism at the Apollo landing sites. Geochim. Cosmochim. Acta 266, 29–53 (2019).

[60] B. Bultel, S. C. Werner, Sample-based spectral mapping around landing sites on the Moon—Lunar timescale part 1. Planet. Sci. J. 4, 146 (2023).

[61] W. Iqbal, H. Hiesinger, C. H. van der Bogert, Geological mapping and chronology of lunar landing sites: Apollo 11. Icarus 333, 528–547 (2019).

[62] H. H. Schmitt, N. E. Petro, R. A. Wells, M. S. Robinson, B. P. Weiss, C. M. Mercer, Revisiting the field geology of Taurus–Littrow. Icarus 298, 2–33 (2017).

[63] W. Iqbal, H. Hiesinger, C. van der Bogert, “New geological maps and crater size-frequency distribution measurements of the Apollo 17 landing site,” in the *50th Lunar and Planetary Science Conference* (Lunar and Planetary Institute, 2019).

[64] R. F. Dymek, A. L. Albee, A. A. Chodos, “Petrology and origin of Boulder #2 and #3, Apollo 17 Station 2,” in the *7th Lunar Science Conference* (Lunar and Planetary Institute/Pergamon Press, 1976).

[65] S. R. Winzer, D. F. Nava, P. J. Schuhmann, R. K. L. Lum, S. Schuhmann, M. M. Lindstrom, D. J. Lindstrom, J. A. Philpotts, The Apollo 17 “melt sheet”: Chemistry, age and Rb/Sr systematics. Earth Planet. Sci. Lett. 33, 389–400 (1977).

[66] D. Stöffler, G. Ryder, B. A. Ivanov, N. A. Artemieva, M. J. Cintala, R. A. F. Grieve, Cratering history and lunar chronology. Rev. Mineral. Geochem. 60, 519–596 (2006).

[67] P. D. Spudis, G. Ryder, “Apollo 17 impact melts and their relation to the Serenitatis basin” in *Multi-ring basins: Formation and Evolution* (Pergamon Press, 1980).

[68] P. D. Spudis, D. E. Wilhelms, M. S. Robinson, The Sculptured Hills of the Taurus Highlands: Implications for the relative age of Serenitatis, basin chronologies and the cratering history of the Moon. J. Geophys. Res. Planets 116, E00H03 (2011).

[69] C. M. Mercer, K. E. Young, J. R. Weirich, K. V. Hodges, B. L. Jolliff, J. A. Wartho, M. C. van Soest, Refining lunar impact chronology through high spatial resolution ^40^Ar/^39^Ar dating of impact melts. Sci. Adv. 1, e1400050 (2015). 26601128 10.1126/sciadv.1400050 PMC4644078

[70] D. Beaty, A. Albee, “Comparative petrology of the Apollo 11 high-K basalts,” in the *9th Lunar Science Conference* (Lunar and Planetary Institute, 1978).

[71] E. A. Jerde, G. A. Snyder, L. A. Taylor, L. Yun-Gang, R. A. Schmitt, The origin and evolution of lunar high-Ti basalts: Periodic melting of a single source at Mare Tranquillitatis. Geochim. Cosmochim. Acta 58, 515–527 (1994).

[72] M. I. Staid, C. M. Pieters, J. W. Head III, Mare Tranquillitatis: Basalt emplacement history and relation to lunar samples. J. Geophys. Res. Planets 101, 23213–23228 (1996).

[73] G. Neukum, P. Horn, Effects of lava flows on lunar crater populations. The Moon 15, 205–222 (1976).

[74] J. Rhodes, J. Brannon, K. Rodgers, D. Blanchard, M. Dungan, “Chemistry of Apollo 12 mare basalts—Magma types and fractionation processes,” in the *8th Lunar Science Conference* (Lunar and Planetary Institute, 1977).

[75] L. E. Nyquist, J. L. Wooden, C. Y. Shih, H. Wiesmann, B. M. Bansal, Isotopic and REE studies of lunar basalt 12038: Implications for petrogenesis of aluminous mare basalts. Earth Planet. Sci. Lett. 55, 335–355 (1981).

[76] J. F. Snape, A. A. Nemchin, J. J. Bellucci, M. J. Whitehouse, R. Tartèse, J. J. Barnes, M. Anand, I. A. Crawford, K. H. Joy, Lunar basalt chronology, mantle differentiation and implications for determining the age of the Moon. Earth Planet. Sci. Lett. 451, 149–158 (2016).

[77] W. Iqbal, H. Hiesinger, C. H. van der Bogert, Geological mapping and chronology of lunar landing sites: Apollo 12. Icarus 352, 113991 (2020).

[78] A. P. Vinogradov, “Preliminary data on lunar ground brought to Earth by automatic probe,” in the *2nd Lunar Science Conference* (Pergamon Press, 1971).

[79] G. Kurat, A. Kracher, K. Keil, R. Warner, M. Prinz, “Composition and origin of Luna 16 aluminous mare basalts,” in the *7th Lunar Science Conference* (Lunar and Planetary Institute/Pergamon Press, 1976).

[80] J. C. Huneke, F. A. Podosek, G. J. Wasserburg, Gas retention and cosmic-ray exposure ages of a basalt fragment from Mare Fecunditatis. Earth Planet. Sci. Lett. 13, 375–383 (1972).

[81] D. A. Papanastassiou, G. J. Wasserburg, Rb-Sr age of a Luna 16 basalt and the model age of lunar soils. Earth Planet. Sci. Lett. 13, 368–374 (1972).

[82] B. A. Cohen, G. A. Snyder, C. M. Hall, L. A. Taylor, M. A. Nazarov, Argon-40-argon-39 chronology and petrogenesis along the eastern limb of the Moon from Luna 16, 20 and 24 samples. Meteorit. Planet. Sci. 36, 1345–1366 (2001).

[83] V. A. Fernandes, R. Burgess, Volcanism in Mare Fecunditatis and Mare Crisium: Ar-Ar age studies. Geochim. Cosmochim. Acta 69, 4919–4934 (2005).

[84] M. A. Ivanov, J. W. Head, H. Hiesinger, New insights into the regional and local geological context of the Luna 16 landing site. Icarus 400, 115579 (2023).

[85] Z. Yue, S. Sun, J. Du, S. Gou, K. Di, Y. Wang, Y. Lin, X. Li, F. Wu, New insights into the geological evolution history of Mare Fecunditatis. Icarus 425, 116348 (2025).

[86] L. S. Tarasov, M. A. Nazarov, I. D. Shevaleevskii, A. F. Kudriashova, A. S. Gaverdovskaia, M. I. Korina, “Mineralogy and petrography of lunar rocks from Mare Crisium (preliminary data),” in the *8th Lunar Science Conference* (Lunar and Planetary Institute, 1977).

[87] A. L. Graham, R. V. Hutchison, Mineralogy and petrology of fragments From the Luna 24 core. Philos. Trans. R. Soc. London Ser. A Math Phys. Eng. Sci. 297, 15–22 (1980).

[88] A. Stettler, F. Albarède, ^39^Ar-^40^Ar systematics of two millimeter-sized rock fragments from Mare Crisium. Earth Planet. Sci. Lett. 38, 401–406 (1978).

[89] O. Schaeffer, A. Bence, J. Papike, D. Vaniman, “^39^Ar-^40^Ar and petrologic study of Luna 24 samples 24077,13 and 24077,63,” in the *9th Lunar Science Conference* (Lunar and Planetary Institute, 1978).

[90] R. Burgess, G. Turner, Laser argon-40-argon-39 age determinations of Luna 24 mare basalts. Meteorit. Planet. Sci. 33, 921–935 (1998).

[91] P. R. Renne, G. Balco, K. R. Ludwig, R. Mundil, K. Min, Response to the comment by W. H. Schwarz et al. on “Joint determination of ^40^K decay constants and ^40^Ar^∗^/^40^K for the Fish Canyon sanidine standard, and improved accuracy for ^40^Ar/^39^Ar geochronology” by P. R. Renne et al. (2010). Geochim. Cosmochim. Acta 75, 5097–5100 (2011).

[92] G. Neukum, B. König, J. Arkani-Hamed, A study of lunar impact crater size-distributions. The Moon 12, 201–229 (1975).

[93] F. K. Aitken, R. Brett, N. J. Hubbard, D. S. Mckay, C. P. Meyer, D. A. Morrison, E. Schonfeld, H. Takeda, “Mineralogy, chemistry, and origin of the KREEP component in soil samples from the Ocean of Storms,” in the *2nd Lunar Science Conference* (Pergamon Press, 1971).

[94] A. C. Stadermann, B. L. Jolliff, M. J. Krawczynski, C. W. Hamilton, J. J. Barnes, Analysis and experimental investigation of Apollo sample 12032,366-18, a chemically evolved basalt from the Moon. Meteorit. Planet. Sci. 57, 794–816 (2022).

[95] S. B. Simon, J. J. Papike, Petrology of the Apollo 12 highland component. J. Geophys. Res. Solid Earth 90, 47–60 (1985).

[96] P. Eberhardt, J. Geiss, N. Grögler, A. Stettler, How old is the crater copernicus? The Moon 8, 104–114 (1973).

[97] E. C. Alexander Jr., A. Bates, M. R. Coscio Jr., J. C. Dragon, V. R. Murthy, R. O. Pepin, T. R. Venkatesan, “K/Ar dating of lunar soils II,” in the *7th Lunar Science Conference* (Lunar and Planetary Institute/Pergamon Press, 1976).

[98] D. D. Bogard, D. H. Garrison, C. Y. Shih, L. E. Nyquist, ^39^Ar-^40^Ar dating of two lunar granites: The age of Copernicus. Geochim. Cosmochim. Acta 58, 3093–3100 (1994).

[99] G. Neukum, B. Koenig, “Dating of individual lunar craters,” in the *7th Lunar Science Conference* (Lunar and Planetary Institute/Pergamon Press, 1976).

[100] K. A. Howard, Avalanche mode of motion: Implications from lunar examples. Science 180, 1052–1055 (1973). 17806579 10.1126/science.180.4090.1052

[101] R. Arvidson, R. Drozd, E. Guinness, C. Hohenberg, C. Morgan, R. Morrison, V. Oberbeck, “Cosmic ray exposure ages of Apollo 17 samples and the age of Tycho,” in the *7th Lunar Science Conference* (Lunar and Planetary Institute/Pergamon Press, 1976).

[102] B. K. Lucchitta, Crater clusters and light mantle at the Apollo 17 site; A result of secondary impact from Tycho. Icarus 30, 80–96 (1977).

[103] E. W. Wolfe, B. K. Lucchitta, V. S. Reed, G. E. Ulrich, A. G. Sanchez, “Geology of the Taurus-Littrow valley floor,” in the *6th Lunar Science Conference* (Lunar and Planetary Institute, 1975).

[104] R. J. Drozd, C. M. Hohenberg, C. J. Morgan, F. A. Podosek, M. L. Wroge, “Cosmic-ray exposure history at Taurus-Littrow,” in the *8th Lunar Science Conference* (Lunar and Planetary Institute, 1977).

[105] K. Marti, B. Lightner, T. Osborn, “Krypton and xenon in some lunar samples and the age of North Ray Crater,” in the *4th Lunar Science Conference* (Pergamon Press, 1973).

[106] C. Behrmann, G. Crozaz, R. Drozd, C. Hohenberg, C. Ralston, R. Walker, D. Yuhas, “Cosmic-ray exposure history of North Ray and South Ray material,” in the *4th Lunar Science Conference* (Pergamon Press, 1973).

[107] R. J. Drozd, C. M. Hohenberg, C. J. Morgan, C. Ralston, Cosmic-ray exposure history at the Apollo 16 and other lunar sites. Lunar surface dynamics. Geochim. Cosmochim. Acta 38, 1625–1642 (1974).

[108] R. Arvidson, G. Crozaz, R. J. Drozd, C. M. Hohenberg, C. J. Morgan, Cosmic ray exposure ages of features and events at the Apollo landing sites. The Moon 13, 259–276 (1975).

[109] H. J. Moore, J. M. Boyce, D. A. Hahn, Small impact craters in the lunar regolith—Their morphologies, relative ages, and rates of formation. Moon Planets 23, 231–252 (1980).

[110] R. A. F. Grieve, M. R. Dence, The terrestrial cratering record: II. The crater production rate. Icarus 38, 230–242 (1979).

[111] Y. Qian, L. Xiao, S. Yin, M. Zhang, S. Zhao, Y. Pang, J. Wang, G. Wang, J. W. Head, The regolith properties of the Chang’e-5 landing region and the ground drilling experiments using lunar regolith simulants. Icarus 337, 113508 (2020).

[112] Q.-L. Li, Q. Zhou, Y. Liu, Z. Xiao, Y. Lin, J.-H. Li, H.-X. Ma, G.-Q. Tang, S. Guo, X. Tang, J.-Y. Yuan, J. Li, F.-Y. Wu, Z. Ouyang, C. Li, X.-H. Li, Two-billion-year-old volcanism on the Moon from Chang’e-5 basalts. Nature 600, 54–58 (2021). 34666338 10.1038/s41586-021-04100-2 PMC8636262

[113] X. Che, A. Nemchin, D. Liu, T. Long, C. Wang, M. D. Norman, K. H. Joy, R. Tartese, J. Head, B. Jolliff, J. F. Snape, C. R. Neal, M. J. Whitehouse, C. Crow, G. Benedix, F. Jourdan, Z. Yang, C. Yang, J. Liu, S. Xie, Z. Bao, R. Fan, D. Li, Z. Li, S. G. Webb, Age and composition of young basalts on the Moon, measured from samples returned by Chang’e-5. Science 374, 887–890 (2021). 34618547 10.1126/science.abl7957

[114] Z. Yue, K. Di, W. Wan, Z. Liu, S. Gou, B. Liu, M. Peng, Y. Wang, M. Jia, J. Liu, Z. Ouyang, Updated lunar cratering chronology model with the radiometric age of Chang’e-5 samples. Nat. Astron. 6, 541–545 (2022).

[115] M. Jia, Z. Yue, K. Di, B. Liu, J. Liu, G. Michael, A catalogue of impact craters larger than 200 m and surface age analysis in the Chang’e-5 landing area. Earth Planet. Sci. Lett. 541, 116272 (2020).

[116] Y. Qian, L. Xiao, S. Y. Zhao, J. N. Zhao, J. Huang, J. Flahaut, M. Martinot, J. W. Head, H. Hiesinger, G. X. Wang, Geology and scientific significance of the Rümker region in northern Oceanus Procellarum: China’s Chang’E-5 landing region. J. Geophys. Res. Planets 123, 1407–1430 (2018).

[117] B. Wu, J. Huang, Y. Li, Y. Wang, J. Peng, Rock abundance and crater density in the candidate Chang’E-5 landing region on the Moon. J. Geophys. Res. Planets 123, 3256–3272 (2018).

[118] Y. Qian, L. Xiao, J. W. Head, C. H. van der Bogert, H. Hiesinger, L. Wilson, Young lunar mare basalts in the Chang’e-5 sample return region, northern Oceanus Procellarum. Earth Planet. Sci. Lett. 555, 116702 (2021).

[119] F. Luo, Z. Xiao, Y. Wang, Y. Ma, R. Xu, S. Wang, M. Xie, Y. Wu, Q. Deng, P. Ma, The production population of impact craters in the Chang’E-6 landing mare. Astrophys. J. Lett. 974, L37 (2024).

[120] Z. Yue, S. Gou, S. Sun, W. Yang, Y. Chen, Y. Wang, H. Lin, K. Di, Y. Lin, X. Li, F. Wu, Geological context of the Chang’e-6 landing area and implications for sample analysis. Int. J. Hydrogen Energ. 5, 100663 (2024). 10.1016/j.xinn.2024.100663 PMC11283046 39071219

[121] I. Garrick-Bethell, K. Miljković, H. Hiesinger, C. H. van der Bogert, M. Laneuville, D. L. Shuster, D. G. Korycansky, Troctolite 76535: A sample of the Moon’s South Pole-Aitken basin? Icarus 338, 113430 (2020).

[122] H. Hiesinger, C. van der Bogert, J. Pasckert, N. Schmedemann, M. Robinson, B. Jolliff, N. Petro, “New crater size-frequency distribution measurements of the South Pole-Aitken basin,” in the *43rd Lunar and Planetary Science Conference* (Lunar and Planetary Institute, 2012).

[123] C. I. Fassett, J. W. Head, S. J. Kadish, E. Mazarico, G. A. Neumann, D. E. Smith, M. T. Zuber, Lunar impact basins: Stratigraphy, sequence and ages from superposed impact crater populations measured from Lunar Orbiter Laser Altimeter (LOLA) data. J. Geophys. Res. Planets 117, E00H06 (2012).

[124] C. Orgel, G. Michael, C. I. Fassett, C. H. van der Bogert, C. Riedel, T. Kneissl, H. Hiesinger, Ancient bombardment of the inner solar system: Reinvestigation of the “fingerprints” of different impactor populations on the lunar surface. J. Geophys. Res. Planets 123, 748–762 (2018).

[125] T. Kneissl, G. G. Michael, N. Schmedemann, Treatment of non-sparse cratering in planetary surface dating. Icarus 277, 187–195 (2016).

[126] S. J. Robbins, A new global database of lunar impact craters >1–2 km: 1. Crater locations and sizes, comparisons with published databases, and global analysis. J. Geophys. Res. Planets 124, 871–892 (2019).

[127] R. V. Wagner, E. J. Speyerer, M. S. Robinson, the LROC Team, “New mosaicked data products from the LROC Team,” in the *46th Lunar and Planetary Science Conference* (Lunar and Planetary Institute, 2015).

[128] H. Sato, M. S. Robinson, S. J. Lawrence, B. W. Denevi, B. Hapke, B. L. Jolliff, H. Hiesinger, Lunar mare TiO_2_ abundances estimated from UV/Vis reflectance. Icarus 296, 216–238 (2017).

