# Supplementary material for: Lunar chronology model with the Chang’e-6 farside samples and implications for the early impact history
Source: Sci Adv. 2026 Feb 4;12(6):eady9265. doi: 10.1126/sciadv.ady9265 (PMC12871470; doi:10.1126/sciadv.ady9265)
Supplement: Supplementary file 1 — Supplementary Note 1 Figs. S1 to S7 Legend for code S1 References [file sciadv.ady9265_sm.pdf]

Science Advances
AAAS

Supplementary Materials for

Lunar chronology model with the Chang'e-6 farside samples and implications for the early impact history

Zongyu Yue *et al.*

Corresponding author: Yangting Lin, linyt@mail.iggcas.ac.cn; Kaichang Di, dikc@aircas.ac.cn

Sci. Adv. **12**, eady9265 (2026)
DOI: 10.1126/sciadv.ady9265

The PDF file includes:

Supplementary Note 1
Figs. S1 to S7
Legend for code S1
References

Other Supplementary Material for this manuscript includes the following:

Code S1

Supplementary Note 1: Review of the Calibration Points in Lunar Cratering Chronology Model

(1) Highland (Terrae)

Both the $N(1)$ and age of the lunar highland are highly suspected and was neglected by refs. (*6, 8, 23, 43, 44*). Ref. (*25*) used the Grimaldi Basin to represent the lunar Highlands yet with an age of 4.456 ± 0.04 Ga and $N(1)$ of $27.4 \times 10^{-2} km^{-2}$.

(2) Nectaris Basin (A16)

Apollo 16 (A16) landed in the Cayley Formation within Descartes Formation that was suggested to relate to the deposition of ejecta material from one or several impact basins including Nectaris and Imbrium Basin, and Apollo 16 astronauts certainly sampled the Descartes Formation material that is basically Nectaris ejecta yet redistributed and modified by the later impact event(*45*). Ref. (*1*) proposed that in the Apollo 16 landing site, the thickness of Nectaris Basin ejection material will be up to several hundred meters although it is at depth. The age accumulation of 4.10 ± 0.10 Ga in the Apollo 16 samples was considered as the age of the Nectaris ejecta, while the age accumulation of 3.90 ± 0.10 Ga is assigned to the Descartes Formation(*1*). Ref. (*46*) also admitted the age of the Nectaris Basin is 4.1 Ga. However, ref. (*47*) proposed the age of Nectaris Basin is 3.85 ± 0.05 Ga, ref. (*48*) pointed out that the Cayley materials are no more than 3.92 Ga, i.e., the age of Nectaris Basin is 3.92 ± 0.03 Ga. Subsequent studies by (*49*) did not find any impact melts in Apollo 16 samples older than 3.91 Ga and proposed the age of Nectaris Basin is probably 3.90 ± 0.02 Ga, which is admitted by ref. (*50*) and is close to the result of 3.92 Ga[51]. In summary, the age of Nectaris Basin is highly controversial and not used in later recalibration of the lunar chronology (*6, 25, 43*).

(3) Apennines (A15)

The Apollo 15 (A15) mission landed at the footwalls of the Apennine Mountain front, in the southeastern part of Mare Imbrium. The Apennine Mountain was interpreted to be part of the Imbrium Basin rim structure(*48*) and the age was assigned 3.91 ± 0.10 Ga(*1*). Subsequent studies have supported the view that Apennine Mountain is part of the Imbrium rim and have also suggested that the samples collected by Apollo 15 contain material from Apennine Mountain. However, these studies presented different ages. For example, ref. (*48*) said the Imbrium age is 3.845 ± 0.03 Ga according to the returned KREEP-basalt fragment and impact melt of breccia matrix. Refs. (*8, 22, 44, 50*) proposed the age of Imbrium is 3.85 ± 0.02 Ga while recently(*52*) assigned 3.992 ± 0.012 Ga to the Imbrium impact. However, ref. (*25*) challenged this view and proposed that the same samples would rather date Orientale Basin and not Imbrium Basin. The calibration point is neglected in recalibrating the lunar chronology recently(*25, 43*).

(4) Descartes Formation (A16)

The ages of the Descartes Formation and the Nectaris Basin are often not clearly distinguished in many papers. Ref. (*1*) assigned 3.90 ± 0.10 Ga to Descartes Formation, and ref. (*8*) provided an age of 3.92 ± 0.03 Ga for Descartes Formation. However, ref. (*6*) proposed that the Descartes formation is not Nectaris ejecta, but rather Imbrium ejecta with an age of 3.85 Ga. Ref. (*43*) used 3.866 ± 0.009 Ga for Descartes Formation. Ref. (*25*) pointed out that it is impossible to define a calibration age for this site at the current state of knowledge.

(5) Fra Mauro Formation (A14)

The landing site of Apollo 14 (A14) is near the outer edge of the Fra Mauro Formation,

which was composed by Imbrium Basin ejecta deposits. The samples returned by Apollo 14 comprise a range of impact melt breccia and it was accepted that they were formed originally by material produced by the Imbrium impact (e.g., refs. (*48, 52*)). The Imbrium Basin age is assigned 3.91±0.1 Ga in ref. (*1*), while ref. (*8*) suggested 3.85±0.02 Ga as the age of Fra Mauro Formation or Imbrium Basin and it was adopted in refs. (*6, 22, 23, 43*). However, ref. (*53*) demonstrated that the age of the Fra Mauro Formation or the Imbrium Basin is 3.926±0.003 Ga based on the apatite U-Pb systems. Subsequently, ref. (*54*) provided an age of 3.938 ± 0.004 Ga for the Imbrium Basin based on the U-Pb analysis of calcium phosphates from Apollo 14 sample 14311. Ref. (*52*) further proposed an age of 3.922±0.012 Ga for the Imbrium Basin based on a link between multiple breccias from different Apollo landing sites and this impact event. It can be observed that there is considerable controversy regarding the age of the Imbrium Basin. However, these studies all agree that the material found in Fra Mauro is ejecta from the Imbrium Basin. Yet, this consensus is also refuted in ref. (*55*), which points out the existence of a resurfacing event in the Apollo 14 landing area and suggests that the samples returned are from the upper ejecta of the Orientale Basin instead of the Imbrium Basin. Ref. (*56*) recently interpret the older sample age of ~4.12 Ga likely represents Nectarian material and the ~3.922 Ga is for the Imbrium Basin impact event. However, this debate is not in contradiction with the fact that this point can serve as a control point.

There is also a substantial controversy regarding the $N(1)$ value in this region, primarily due to different counting areas selected, and the maximum discrepancy is nearly four times. Ref. (*56*) summarized and compared the previous results, including those from refs. (*1, 22, 23, 55*). Ref. (*56*) conducted a detailed comparison of these results and analyzed the reasons for the discrepancies among them, and they further employed different datasets to compare and analyze the obtained $N(1)$ values, finding consistent results. Finally, with the fit range of 1.4~3.0 km for the crater diameter, they derived the $N(1)$ is $(3.82 \pm 1.07) \times 10^{-2}$ km^{-2} with

the lunar crater PF of ref. (*1*) or $(3.60 \pm 1.01) \times 10^{-2}$ km^{-2} with the lunar crater PF of ref. (*29*). Therefore, we consider the result of $(3.82 \pm 1.07) \times 10^{-2}$ km^{-2} to be the most reliable and adopted it in this study.

(6) Taurus Littrow Mare (A17)

The landing site of Apollo 17 (A17) was on the dark floor of a marginal embayment of Mare Serenitatis called the Taurus-Littrow Valley at the south edge of the Serenitatis Basin. The returned basalt samples can be classified into three types A, B, and C which are sufficiently different to indicate extrusion as a separate lava flow(*57*). In earlier studies, a total of 18 age determinations by the ^{39}Ar-^{40}Ar method have been carried out on Apollo 17 basalts and the resulted ages range from 3.69 Ga to 3.84 Ga(*58*). Ref. (*48*) accepted the age of 3.72 Ga as the emplacement age of the entire sequence, while ref. (*1*) took the age of 3.70 ± 0.10 Ga as the calibration age for the Apollo 17 site and it is also used in ref. (*22*). Subsequently, ref. (*8*) suggested the Apollo 17 basalt can represent at least four distinct extrusions and most of the samples are group A at the age of 3.75 ± 0.01 Ga, which was adopted by refs. (*6, 43*) as the calibration point for the Mare Serenitatis. Recently, ref. (*59*) measured an age of 3.752 ± 0.007 Ga for the Group A basalt. Ref. (*60*) verified group A basalt samples match the remote-sensing spectral data best. Ref. (*25*) also claimed that they referred to the measured age by ref. (*61*); however, they used the age 3.753 ± 0.009 Ga as the calibration point.

For the crater size-frequency distribution, some studies used that for the Mare Serenitatis(*23*) or did not specify the measured region (e.g., refs. (*6, 43*)). However, recent stratigraphic analysis demonstrates that the Taurus–Littrow valley is underneath the nearby Mare Serenitatis and then older than the latter(*25, 62*), thus it is better to measure the $N(1)$ in local Taurus Littrow valley. Both refs. (*25*) and (*63*) used the Mare Taurus–Littrow as the

counting area, but the sizes of their respective areas are different. In the counting area of ref. (*25*), a region potentially containing secondary craters has been excluded. In this sense, the results from ref. (*25*) are more reliable. However, a substantial uncertainty will exist if the counting area is too small, as shown by the notable deviation of the crater size-frequency distribution from the isochron in ref. (*25*). In contrast, the results of ref. (*63*) align well with the isochron especially with the large craters. Therefore, although a small portion of secondary craters may have contaminated the statistical data, they were actually not used in the calculation of $N(1)$. Consequently, the results of $(1.06 \pm 0.21) \times 10^{-2}$ km^{-2} from ref. (*63*) is more reliable and used in this research. The $N(1)$ is obtained based on the lunar PF of ref. (*1*) and the fit range is about 0.3~0.6 km for the crater diameter.

There are also some impact melts within Apollo 17 samples; however, a notable controversy has existed for long time regarding whether the impact melts are formed during the Serenitatis Basin impact event. Some breccia clasts are interpreted as clast-laden impact melts related to the Serenitatis basin-forming event(*64, 65*), and some studies ascribed the poikilitic samples to be the Serenitatis basin melt sheet and measured an age of 3.89 ± 0.02 Ga(*8, 66*). However, this view was opposed by many studies. For example, ref. (*67*) suggested that the Apollo 17 highland melt breccias are not all derived from the Serenitatis basin impact; specifically, the aphanitic melt rocks may be either other basin or local crater ejecta. Ref. (*68*) further pointed out that the returned impact breccia is a distal facies of Imbrium basin ejecta and is not directly related to the Serenitatis basin-forming impact. Recently, ref. (*69*) measured the Apollo 17 impact melt breccias with new laser microprobe $^{40}Ar/^{39}Ar$ data and found a single melt-forming event at about 3.83 Ga, while data from the other sample document multiple impact melt–forming events between ca. 3.81 Ga and at least as young as ca. 3.27 Ga. They concluded that Apollo 17 impact breccia samples should include a substantial component of ejecta from the Imbrium basin impact. In summary, the age of Serenitatis Basin is poorly

constrained and it is seldom used as the calibration point.

(7) Mare Tranquillitatis (A11)

The Apollo 11 (A11) landing site is located at the southwestern edge of Mare Tranquillitatis and two groups, with one of high-K (group A) and the other low-K (group B) are classified in earlier studies(*70*). Ref. (*58*) collected the measured radiometric ages for the two groups of basalts, based on which ref. (*1*) assigned the ages of 3.72 ± 0.10 and 3.53 ± 0.05 Ga to the group B and group A basalts. In subsequent research, the Apollo 11 basalts were further subdivided in detail. Five groups of basalts (A, Bl, B2, B3, D), ranging from the low-K, low rare-earth element (REE) (Groups Bl, B2, and B3) to the low-K, high- REE Group D basalts, to the high-K, high-REE Group A basalts, are classified(*71*). The group A is the youngest with an age of 3.59 ± 0.02 Ga(*71*), and refs. (*8, 72*) provided an age of 3.58 ± 0.01 Ga based on the data compiled from various sources and it was used in many recent studies in calibrating lunar chronology function(*6, 22*). Ref. (*43*) also referred to ref. (*8*); however, they used 3.60 ± 0.01 Ga. Most recent radiometric measurement of the group A basalt is from ref. (*61*), which provided an age of 3.578 ± 0.009 Ga from Pb-Pb isochrons generated using Secondary Ion Mass Spectrometry (SIMS) analyses and it was accepted in ref. (*25*). We decided to use the age of 3.578 ± 0.009 Ga as it is obtained with the most reliable method.

The *N*(1) value in the landing area has also been measured in many studies. Ref. (*73*) found there are fine breaks in the slope of the crater size-frequency distribution of the craters in Apollo 11 landing area, and they said there are two possible associations of the crater frequencies with radiometric ages of the Apollo basalt samples. This is probably the basis for the two calibration points for Mare Tranquillitatis in ref. (*1*), despite the difference in data sets

used between the two documents. However, recent studies (see refs. (*23, 25, 61*)) only find a single isochron. In fact, the ages of older basalt are rarely used when updating the chronological function in subsequent studies. With the fit range of 0.25~0.90 km for crater diameter, ref. (*61*) obtained $N(1)$ is $(6.64 \pm 0.561) \times 10^{-3}$ km^{-2} (Note: there is a typo in the paper) with the lunar crater PF of ref. (*1*) or $(6.42 \pm 0.543) \times 10^{-3}$ km^{-2} with the lunar crater PF of ref. (*29*). Ref. (*61*) compared the results using different counting areas and different images for analysis and their results are reliable.

(8) Mare Imbrium (A15)

The basalts returned by Apollo 15 (A15) are divided into four types based on their texture and mineralogy in early studies, and the median age is 3.28 Ga(*58*). This age was subsequently adopted by ref. (*1*). Later research usually classified the basalts into two groups of the olivine-normative basalts (3.30 Ga) and the quartz-normative basalts (3.35 Ga), and ref. (*8*) proposed the crater density measured in the landing area can be ascribed to an age of 3.30 Ga with confidence. The age is adopted in recent studies on lunar cratering chronology function(*6, 22, 43*). The most recent radiometric measurements are from ref. (*59*), and ref. (*25*) used the average (3.281 ± 0.012 Ga) of the ages of olivine-normative basalts for calibration.

The $N(1)$ value of the Apollo 15 landing area was assigned $(3.2 \pm 1.1) \times 10^{-3}$ km^{-2} in ref. (*1*), which is close to the result of 2.6×10^{-3} km^{-2} from ref. (*48*). Ref. (*23*) provide a much higher $N(1)$ of $(5.50 \pm 1.34) \times 10^{-3}$ km^{-2} and it is close to the result of 5.468×10^{-3} km^{-2} reported in ref. (*22*) that is derived using the near-Earth objects (NEO) and main belt asteroids (MBA) size distributions. However, ref. (*25*) indicated that the counting area by ref. (*23*) is compositional differences and includes some large craters that are obviously earlier than the lava flows, which resulted in the doubled crater-frequency value. The most recent $N(1)$

result of $(2.23 \pm 0.12) \times 10^{-3}$ km^{-2} in Apollo 15 landing area is obtained by ref. (*25*), in which the counting area is spectrally homogeneous and morphologically supported unit(*60*). The lunar crater PF used to derive $N(1)$ is from ref. (*29*), and the fit range is about 0.2~0.8 km for the crater diameter.

(9) Oceanus Procellarum (A12)

Apollo 12 (A12) landed in Mare Cognitum located in the southeastern Oceanus Procellarum, and the returned basalts can be classified into four groups: the olivine, pigeonite, ilmenite, and feldspathic basalts respectively(*74*). Ref. (*58*) summarized the radiometric measured sample ages ranging from 3.08 to 3.24 Ga, and ref. (*1*) assigned the age of 3.18 ± 0.10 Ga for the age. Ref. (*8*) summarized the absolute ages of the landing area and suggested it is 3.30 ± 0.02 Ga for Apollo 12 landing area. The age is used in refs. (*6, 22, 43*). Recent studies show that the feldspathic-basalt group spectra match the remote-sensing spectral signature best at and around the Apollo 12 landing site(*60*). For the feldspathic basalt sample, the crystallization age has been measured at 3.35 ± 0.09 Ga(*75*) and 3.20 ± 0.08 Ga(*8*). The most recent measured age of the Apollo 12 feldspathic basalt sample is 3.242 ± 0.013 Ga(*76*), and it is used in ref. (*25*). We also used it as the calibration point considering it is the compositional matched with the remote sensing observation.

Ref. (*73*) mapped the craters surrounding the Apollo 12 landing site and found a distinct break at diameter ~1.3 km in the crater size-frequency distribution. They concluded that the population of smaller craters relates to the returned samples, and they assigned $N(1)$ 2.5×10^{-3} km^{-2}. However, ref. (*1*) adjusted it to be $(3.6 \pm 1.1) \times 10^{-3}$ km^{-2}. Ref. (*23*) defined determined $N(1)$ as $(5.9 \pm 0.9) \times 10^{-3}$ km^{-2} within a different counting area. However, ref. (*77*) found the counting area selected by ref. (*23*) consists of several older mare

flows, which is probably the reason for his higher result. Ref. (*77*) provided the *N*(1) of 2.81×10^{-3} km^{-2} by selecting two homogenous areas; however, the mapping area is so small that only 7.6 craters are used to derive the *N*(1). The most recent result is from ref. (*25*), in which the counting area is carefully selected based on the spectral homogeneity and the crater size-frequency distribution is well consistent with the isochron. The lunar crater PF used to derive *N*(1) is from ref. (*29*), and the fit range is about 0.2~0.9 km for the crater diameter. We thus used the result of $(2.34 \pm 0.05) \times 10^{-3}$ km^{-2} in this study.

(10) Mare Fecunditatis (L16)

Luna 16 (L16) landed in northeast of the Mare Fecunditatis and returned ~101 g drill-core samples(*78*), and the returned sample is primarily a unique type of high-alumina basalt(*79*). Earlier radiometric measurements indicating the age of the igneous activity in Mare Fecunditatis is 3.45 ± 0.04 Ga(*80*), 3.42 ± 0.18 Ga(*81*), while ref. (*58*) provided two ages of 3.41 ± 0.04 Ga and 3.35 ± 0.18 Ga. Ref. (*1*) assigned 3.40 ± 0.04 Ga as the age of Mare Fecunditatis. Ref. (*8*) provide the age of 3.41 ± 0.04 Ga as the age of Mare Fecunditatis and it was used in subsequent studies in establishing the lunar chronology function(*6, 22, 43*). Recent measurements by ref. (*82*) showed two episodes of 3.347 ± 0.024 Ga and 3.421 ± 0.030 Ga, while ref. (*83*) found a single period of volcanic activity with the age of 3.37 ± 0.02 Ga or 3.35 ± 0.04 Ga. Based on the results of ref. (*82*), ref. (*25*) selected the average age of 3.382 ± 0.014 Ga as the calibration point, and we also used it in this study.

Ref. (*1*) provided the *N*(1) of $(3.3 \pm 1.0) \times 10^{-3}$ km^{-2} as the crater frequency in the Luna 16 sampling area, ref. (*23*) obtained a result of $(5.82 \pm 0.64) \times 10^{-3}$ km^{-2} within a much larger counting area, and ref. (*84*) provided the result of $(4.20 \pm 0.13) \times 10^{-3}$ km^{-2} within a similar counting area. However, ref. (*25*) said that the mapped craters in ref. (*23*)

includes several lava embayed large craters to be relics of incomplete lava emplacement, which is probably the reason that such a higher results obtained. Ref. (*25*) obtained the result of $(2.46 \pm 0.09) \times 10^{-3}$ km^{-2}; however, in their results, these data points always deviate from the isochron more or less, potentially due to the relatively small statistical area. In this research we adopted the $N(1)$ of $(4.32 \pm 0.01) \times 10^{-3}$ km^{-2} from the most recently obtained by ref. (*85*), during which the counting area is spectral and topographic homogeneous and the Lunar Reconnaissance Orbiter Camera (LROC) Narrow Angle Camera (NAC) images are used in mapping craters. The fit range is about 0.37~2.8 km for crater diameter and the lunar crater PF is from ref. (*29*).

(11) Mare Crisium (L24)

Luna 24 (L24) landed on the southeast of Mare Crisium and returned 170 g drill core high-Al_2O_3, low-TiO_2 basalts samples(*86, 87*). Earlier radiometric measurements with the ^{39}Ar-^{40}Ar technique indicated the lava flows were extruded at 3.65 ± 0.12 Ga(*88*), 3.33 ± 0.21 Ga(*89*), and 3.2~3.6 Ga(*58*), while ref. (*1*) used the age of 3.30 ± 0.10 Ga as the calibration point. Later measurement result by ref. (*90*) is 3.22 ± 0.04 Ga, while ref. (*8*) used the age of 3.22 ± 0.02 Ga and it was adopted in refs. (*6, 22, 43*). In addition, ref. (*82*) found the Luna 24 very low-Ti basalt fragments have a unimodal age distribution of 3.273 ± 0.083 Ga, indicating that the returned samples come from a single extrusive episode within Mare Crisium. Ref. (*25*) suggested the average age of 3.328 ± 0.021 Ga for the calibration after considering the updated ^{40}K decay constant of ref. (*91*).

Ref. (*92*) first mapped the craters in the eastern part of Mare Crisium and derived $N(1)$ value of 2.63×10^{-3} km^{-2}, and later ref. (*1*) reported $N(1)$ $(3.0 \pm 0.6) \times 10^{-3}$ km^{-2} in establishing the lunar chronology function. Ref. (*23*) derived $N(1)$ as $(4.66 \pm 0.58) \times 10^{-3}$

km^{-2}; however, ref. (*25*) found that many large craters older than the lava emplacement are included in his results. Based on spectral analysis(*60*), ref. (*25*) obtained the $N(1)$ as $(2.54 \pm 0.08) \times 10^{-3}$ km^{-2} surrounding the Luna 24 landing site. The result is largely consistent with that in ref. (*1*), and it is used in this research. The fit range is 0.2 km~1.6 km for the crater diameter, and the lunar crater PF is from ref. (*29*).

(12) Copernicus (A12)

Apollo 12 landing site is near a ray associated with the crater Copernicus, which is situated approximately 370 km to the north. The returned samples are mainly mare basalts, with some regolith breccias consisting of ropy glasses and impact melt fragments that are widely considered as Copernicus ejecta(*8, 48, 93-95*). With the $^{39}Ar/^{40}Ar$ method, the KREEP glass samples are dated 0.80 ± 0.04 Ga, and it is considered as the age of Copernicus crater(*96, 97*). Ref. (*58*) provided an age of ~0.85 Ga for Copernicus crater and it is accepted by ref. (*1*). Other measurements indicate that the age of the Copernicus crater is 0.80 ± 0.015 Ga(*8, 98*), and it is accepted in subsequent lunar chronology function(*6, 22*). In addition, ref. (*43*) used 0.78 ± 0.015 Ga as the calibration point yet without providing the reference. In this research, we adopted 0.80 ± 0.015 Ga for the calibration.

The crater frequency data were measured on the interior and ejecta of Copernicus crater(*99*), based on which ref. (*1*) used $(1.3 \pm 0.3) \times 10^{-3}$ km^{-2} for calibration. The value was adopted in subsequent studies(*8, 46*). Using the high-resolution orbital images, ref. (*24*) measured the crater size-frequencies of the areas from impact melt pools, crater floor, continuous ejecta blanket of Copernicus crater and the bright ray area north of the Apollo 12 landing site. They prefer the results obtained on the ejecta blanket, and $N(1)$ of 6.68×10^{-4} km^{-2} was provided based on all the ejecta blankets with 19615 craters, which gives the

uncertainty of 0.048×10^{-4} km^{-2}. Ref. (*43*) also referred to the result of ref. (*24*), yet they used $(6.67 \pm 0.527) \times 10^{-4}$ km^{-2} as the *N*(1) value. In addition, ref. (*6*) provided the *N*(1) of 7.15×10^{-4} km^{-2}, and ref. (*22*) derived the *N*(1) values of 1.321×10^{-3} km^{-2} and 1.348×10^{-3} km^{-2} based on the NEO and MBA size distributions for the Copernicus ejecta and floor, respectively. The Copernicus crater was not used in the chronology work in ref. (*23*), because it was considered as an indirectly determined absolute age therein. In this research we also adopted the result from ref. (*24*), in which the fit range is 0.07~0.30 km and the lunar crater PF from ref. (*29*) was used.

(13) Tycho (A17)

A number of investigators have suggested that the Apollo 17 (A17) landing site are related to impact of ejecta from the crater Tycho, although which lies some 2000 km to the southwest(*100-103*). Consequently, the cosmic ray exposure ages of Apollo 17 samples can be interpreted to represent the formation age of Tycho crater. The exposure age of Apollo 17 samples has been dated at 0.096 ± 0.005 Ga(*101*) or 0.109 ± 0.004 Ga(*104*), and the latter is used by ref. (*1*) in establishing the lunar chronology function. The age is also adopted in recent studies for lunar chronology updating(*6, 8, 22, 23, 43*).

Ref. (*1*) reported the *N*(1) as $(9.0 \pm 1.8) \times 10^{-5}$ km^{-2} on the continuous ejecta blanket of Tycho crater, and it is also adopted in ref. (*8*). Ref. (*24*) made crater counts on the floor, impact melt, and the continuous ejecta blanket of Tycho crater. They also preferred the results obtained on the ejecta blanket are more correlated with the returned samples. In their results, the *N*(1) value is 7.12×10^{-5} km^{-2} according to 12698 craters counted in all the Tycho crater ejecta. Thus, the value of $(7.12 \pm 0.063) \times 10^{-5}$ km^{-2} is used in this research. This result is based on the lunar crater PF of ref. (*29*) and the fit range is about 0.017~0.07 km for the crater

diameter.

(14) North Ray (A16)

North Ray crater (diameter: 1.0 km) is one rayed crater nearby Apollo 16 (A16) landing site. The exposure age of the returned samples was measured at 0.0489 ± 0.0017 Ga(*105*), 0.0506 ± 0.0038 Ga(*106*), 0.0503 ± 0.0008 Ga(*107*), etc. Ref. (*1*) claimed to use the result by ref. (*107*) yet they adopted 0.0500 ± 0.0014 Ga as the calibration point, while ref (*8*) used 0.053 ± 0.008 Ga (It seems there is a typo therein and it is followed in recent studies, such as refs (*6, 22, 43*)). Thus we adopted the result from ref. (*107*) in this research.

Based on the crater frequency data on the ejecta, ref. (*1*) obtained the *N*(1) of $(4.4 \pm 1.1) \times 10^{-5}$ km^{-2} for the North Ray crater. The results was adopted in the review papers(*6, 8*). Ref. (*24*) used the same four count areas as defined in the work of ref. (*1*) yet with the high resolution LROC NAC images to update the crater frequency corresponding to the North Ray crater. In this research we adopted the *N*(1) of 3.90×10^{-5} km^{-2} corresponding to 8117 craters therein, i.e., $(3.90 \pm 0.043) \times 10^{-5}$ km^{-2} as the *N*(1) for North Ray crater. This result is based on the lunar crater PF from ref. (*29*) and the fit range is about 0.010~0.045 km for the crater diameter.

(15) Cone (A14)

Cone crater (diameter: 340 m) is located about 1100 m northeast of the Apollo 14 landing site and exhibits a sharp rim. Samples from Cone crater ejecta were collected during the Apollo 14 mission, and their exposure ages can be used to date the formation of Cone crater. The measured result is 0.024 Ga(*107*), 0.026 Ga(*108*), or 0.0244 ± 0.0011 Ga(*106*). Ref. (*1*) adopted 0.0260 ± 0.0008 Ga as the calibration in Cone crater. Ref. (*8*) used $0.0250 \pm$

0.012 Ga in their research, and this result is followed in recent studies(*6, 22, 43*). In this research we adopted the result from ref. (*108*) as the calibration.

The crater frequency of Cone crater was measured on its ejecta blankets(*109*) with the diameter range of about 5m~20 m. Ref. (*1*) used the measurements to obtain the $N(1)$ of $(2.1 \pm 0.5) \times 10^{-5}$ km^{-2} for the Cone crater based on the lunar crater PF of ref. (*1*), and the result is followed by refs. (*8, 46*). The result is also used in the recent studies(*6, 43*) and we also adopted in this research.

(16) Phanerozoic craters (North American and East Europe, lunar equivalent)

Ref. (*110*) estimated the frequency of terrestrial craters for diameter larger than 20 km on the North American and East European cratons, and pointed out that it overlaps that on the Moon if the effects of variations in impact velocity, surface gravity, and gravitational cross section are considered. Ref. (*1*) listed it as a calibration point by converting it for lunar impact conditions and assigned age of the cratons. However, this conversion process involves a great deal of uncertainty(*37*), and it has rarely been used in later studies on updating the lunar cratering chronology function(*6, 8, 23, 43, 44*).

(17) Northern Oceanus Procellarum (CE-5)

The landing site of Chang'e-5 (CE-5) is in Northern Oceanus Procellarum, which belongs to the geologic unit Em4 mapped by ref. (*111*). The age of the returned mare basalt is 2.030 ± 0.004 Ga using the precise lead–lead method(*112*), which is consistent with the result of 1.963 ± 0.057 Ga from ref. (*113*) but with a higher accuracy and is adopted in this research. The age is also used in updating lunar chronology function(*25, 43, 114*).

We have obtained the crater frequency of the CE-5 landing area and obtained $N(1)$ of $(1.74 \pm 0.022) \times 10^{-3}$ km^{-2} based on the crater measurements in a high-resolution orthophoto map, which is generated from more than 700 LROC NAC images(*115*). Other results include $(1.02 \pm 0.026) \times 10^{-3}$ km^{-2} from ref. (*116*), $(1.24 \pm 0.14) \times 10^{-3}$ km^{-2} from ref. (*117*), $(1.28 \pm 0.022) \times 10^{-3}$ km^{-2} from ref. (*118*), and $(2.61 \pm 0.081) \times 10^{-3}$ km^{-2} from ref. (*43*), etc. In this research, we adopted the result of $(1.74 \pm 0.022) \times 10^{-3}$ km^{-2} because the craters are manually mapped in the highest resolution mosaic up to date. This result is based on the lunar crater PF of ref. (*29*) and the fit range is about 0.25~2.0 km for the crater diameter.

(18) Mare Apollo (CE6)

Chang'e-6 (CE-6) landed on the southern mare of the Apollo Basin, located in the northeastern SPA Basin(*18*). The returned samples are primarily a mixture of local basalts and non-basaltic ejecta materials(*18*). The age of the local basalt is measured at 2.807 ± 0.003 Ga(*19*) or 2.830 ± 0.005 Ga(*20*). The two results are relatively consistent and we used 2.807 ± 0.003 Ga(*19*) in this research.

The crater frequency of the CE-6 landing area has been measured in several studies, including $(3.26 \pm 0.092) \times 10^{-3}$ km^{-2} from ref. (*14*), $(2.01 \pm 0.091) \times 10^{-3}$ km^{-2} from ref. (*15*), $(2.47 \pm 0.11) \times 10^{-3}$ km^{-2} from ref. (*16*), $(2.01 \pm 0.90) \times 10^{-3}$ km^{-2} from ref. (*119*). We found that the $N(1)$ in the landing area is closely related to the counting area, and it will initially increase and then remain constant when gradually narrowing the counting area centered on the landing point(*120*). Thus we adopted $(2.08 \pm 0.13) \times 10^{-3}$ km^{-2} as the $N(1)$ in the research. This result is based on the lunar crater PF of ref. (*29*) and the fit range is about 0.25~0.6 km for the crater diameter.

(19) SPA basin (CE-6)

There are also many non-mare materials in the samples returned by CE-6(*18*), and the norites among them is considered as the SPA impact melt mainly based on the mineral characteristics(*21*). The precise age of the SPA basin is measured at 4.247 ± 0.005 Ga with the Pb–Pb method on zirconium-bearing minerals in the norites(*21*). This can be used to constrain the early impact flux. In addition, based on the Apollo 17 sample troctolite 76535, ref. (*121*) also obtained the age of SPA basin is 4.25 ± 0.01 Ga that is remarkably consistent with the result from ref. (*21*). An age of ~4.32–4.33 Ga was derived based on the lunar meteorite Northwest Africa 2995(*28*). Considering the importance of the SPA basin age in analyzing the early lunar impact history, particularly the Late Heavy Bombardment (LHB), this study also attempts to use 4.33 Ga as the age of the SPA basin (**fig. S1**).

The crater frequency of SPA basin has been measured in many studies, and the resulted $N(1)$ are exemplified as 3.70×10^{-1} km^{-2} (ref. (*122*)) and $(3.6 \pm 0.1) \times 10^{-1}$ km^{-2} (ref. (*121*)). Note the $N(1)$ values are fitted with the lunar crater PF of ref. (*1*). For the SPA basin, the frequency of craters larger than 20 km in diameter, i.e., $N(20)$ instead of $N(1)$ is provided in some studies. The value of $N(1)$ can be obtained by multiplying the value of $N(20)$ by a factor of 994.68 if the crater production function from ref. (*1*) is used or by a factor of 419.46 if the crater production function from ref. (*29*) is used. Ref. (*123*) derived the $N(20)$ is larger than $(1.56 \pm 0.07) \times 10^{-4}$ km^{-2} for the SPA basin, yet they did not explicitly list the lunar crater PF used herein. However, ref. (*124*) pointed out that for the large impact basins, the buffered non-sparseness correction (BNSC)(*125*) must be made to account for the loss of craters due to subsequent crater/ejecta coverage. The corrected $N(20)$ is $(2.54 \pm 0.21) \times 10^{-4}$ km^{-2} for the SPA basin(*124*) with the lunar crater PF of ref. (*1*), and the $N(1)$ will be $(2.53 \pm$

$0.21) \times 10^{-1}$ km^{-2}. Unfortunately, the basalt areas are also included in the counting area by ref. (*124*), which is unsuitable because they are much younger than the SPA basin. Therefore, we have refined the $N(1)$ value for SPA basin. The counting area is largely consistent with the rim of SPA basin but the maria basalt areas are excluded (**fig. S1**). The craters mapped by ref. (*126*) are used to calculate the crater frequency. Ref. (*126*) claimed that all the craters larger than 1-2 km in diameter have been extracted. To remove the possible secondary craters, we selected 16371 craters larger than 4.0 km in diameter to calculate the crater frequency. In addition, we do not apply the randomness test to the crater count of SPA because, being a non-sparse distribution (i.e. the area occupied by impact structures is a non-negligible fraction of the counting area), the spatial distribution of smaller craters is skewed by the obliteration of some of their population by larger ones. While the effect on smaller crater density is accounted for by the non-sparseness correction, we do not presently have an appropriate correction for the randomness analysis in this scenario. The obtained $N(1)$ value is $(3.69 \pm 0.48) \times 10^{-1}$ km^{-2} based on the lunar crater PF of ref. (*1*). The fit range is about 80 km~300 km for the crater diameter.

(20) Summary

The lunar cratering chronology function is the foundation in studying the lunar impact history and dating the lunar surface through establishing the relationship between the radiometric ages of the returned lunar samples and the crater frequencies in the corresponding areas. With the technological advancements, the radiometric measurements of samples are becoming more and more accurate. On the other hand, with the improvement of lunar imagery resolution, the measured crater frequencies are also more reliable. It is important to note that the crater frequency of $N(1)$ is usually dependent on the lunar crater PF. We have demonstrated that when the fit range of crater diameter is less than 1 km, the $N(1)$ values obtained by PF

from refs. (*1*) and (*29*) are very close(*114*). Additionally, our review shows that the crater PF from ref. (*1*) is more commonly used when analyzing the crater frequency in the SPA basin. Therefore, we decided to adopt the impact crater production function from ref. (*1*). Table 1 lists the values of these calibration points.

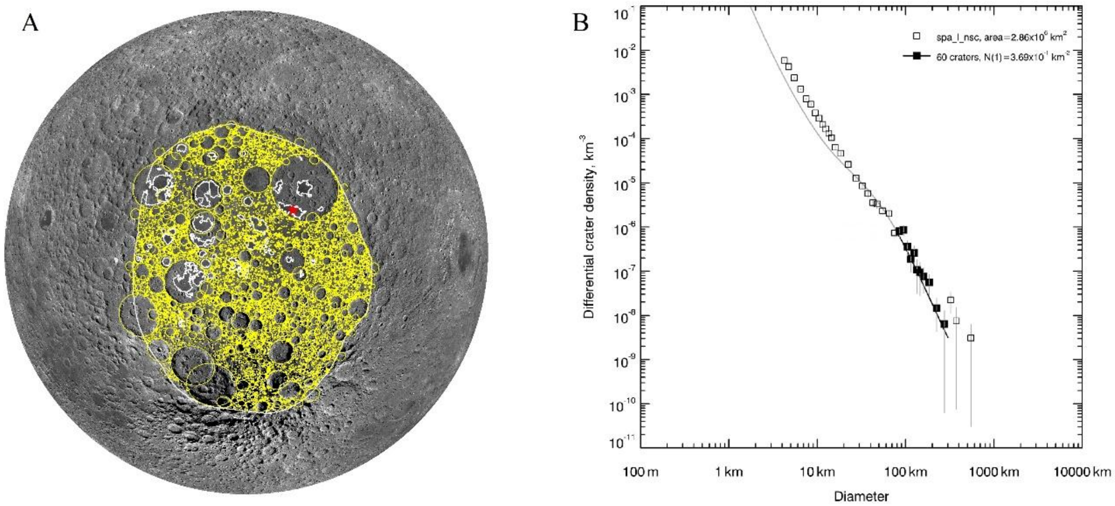

fig. S1. Crater frequency measured at SPA basin. (a) The counting area (white polygons) and mapped craters (yellow circles) in SPA basin. The boundary of the counting area is largely consistent with the rim of SPA basin, while the maria basalt areas within are excluded. The craters (diameter ≥4.0 km) are referred to the lunar crater catalogue compiled by ref. (*23*). The red star is the landing site of the CE-6. (b) The crater size-frequency distribution and derived *N*(1) with the lunar crater PF from ref. (*1*). The fit range as shown by the filled squares is 80 km~300 km for the crater diameter.

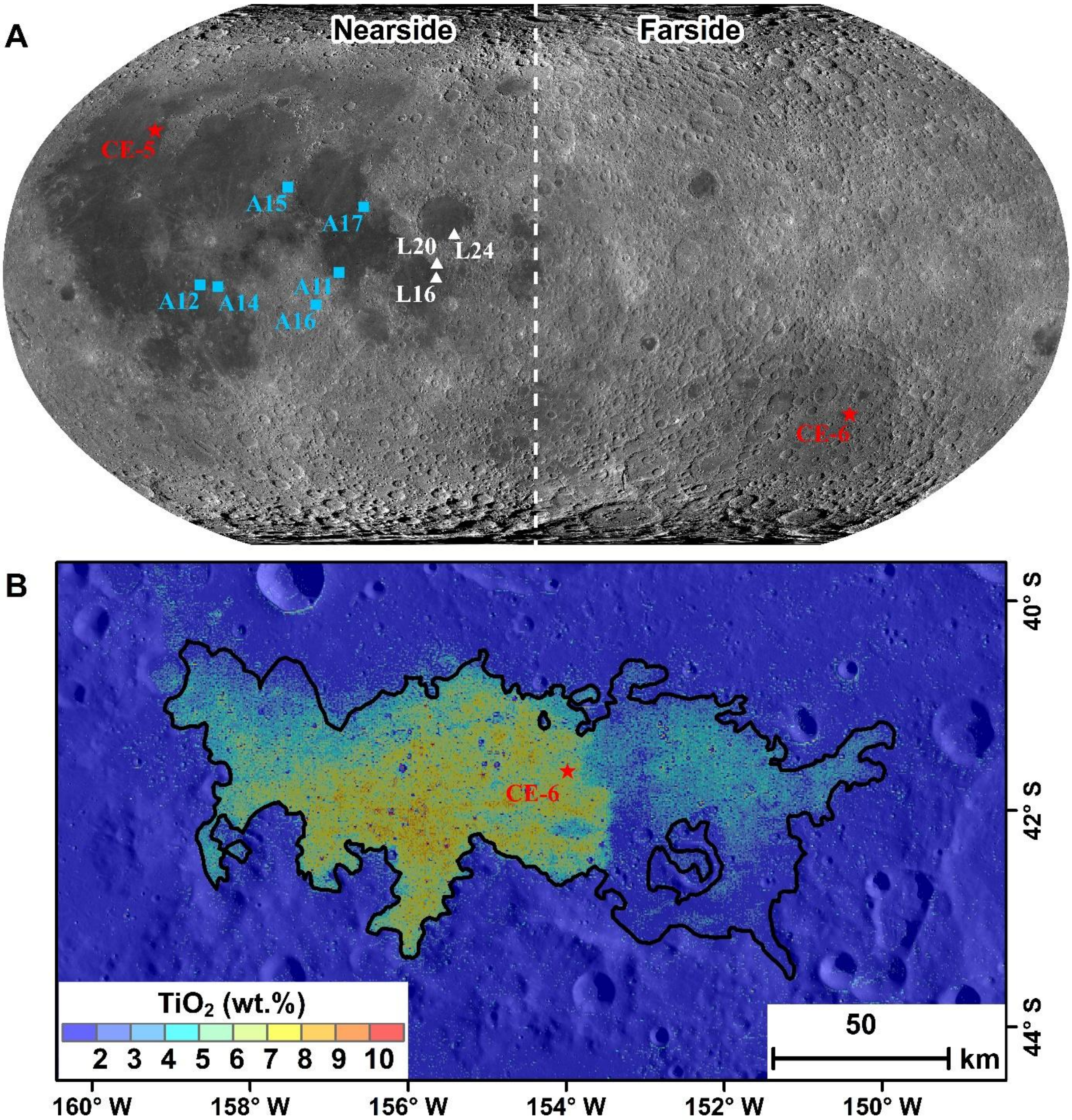

fig. S2. Location of the Chang'e-6 landing site. (a) On the lunar farside, with WAC mosaic(*127*) as the basemap; (b) Inside the Apollo basin within the SPA basin. TiO_2 abundance is from ref. (*128*).

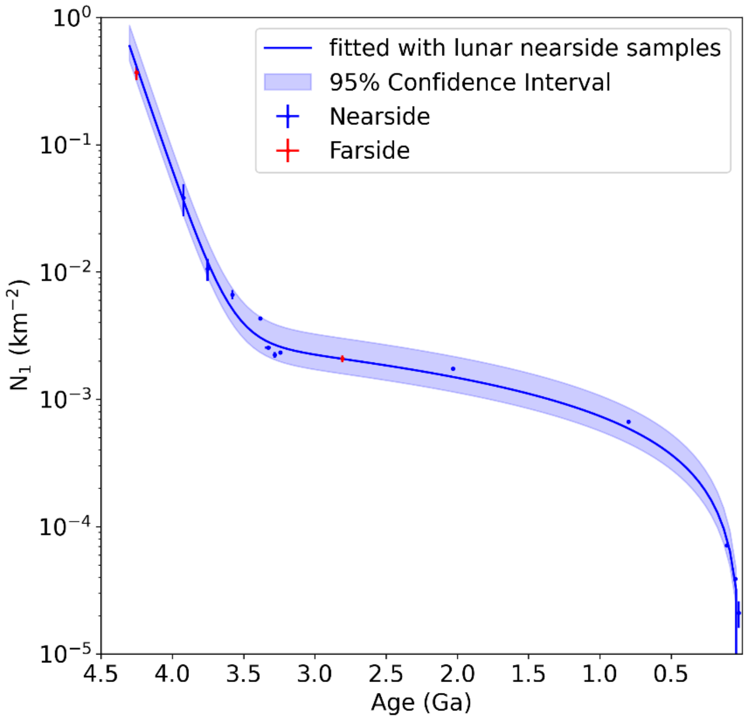

fig. S3. The fitted lunar CF with the data sets of lunar nearside samples compared with the two data sets from CE-6 samples. The blue points represent the data sets of the lunar nearside samples, the blue line is the fitted lunar CF only with the data sets of those samples, and the blue band is the 95% confidence interval of the fitted lunar CF. The two red crosses are the data sets of the lunar farside samples returned by CE-6 mission.

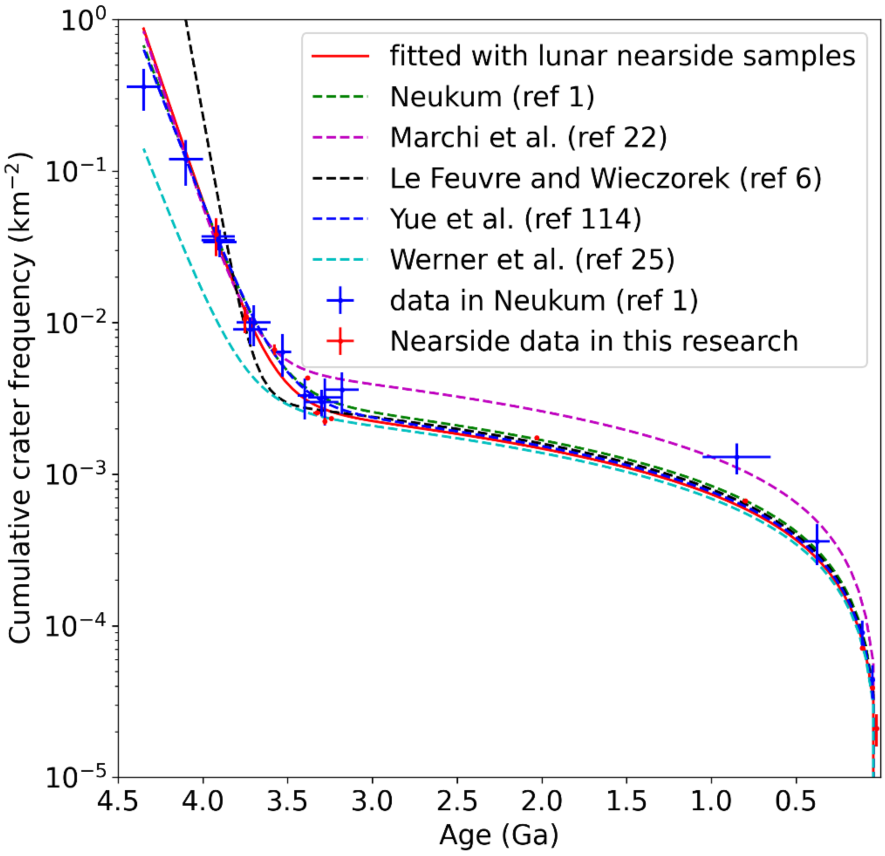

fig. S4. Comparison of the fitted lunar CF only with nearside samples and other models.

Note all the above models are based on the lunar nearside samples.

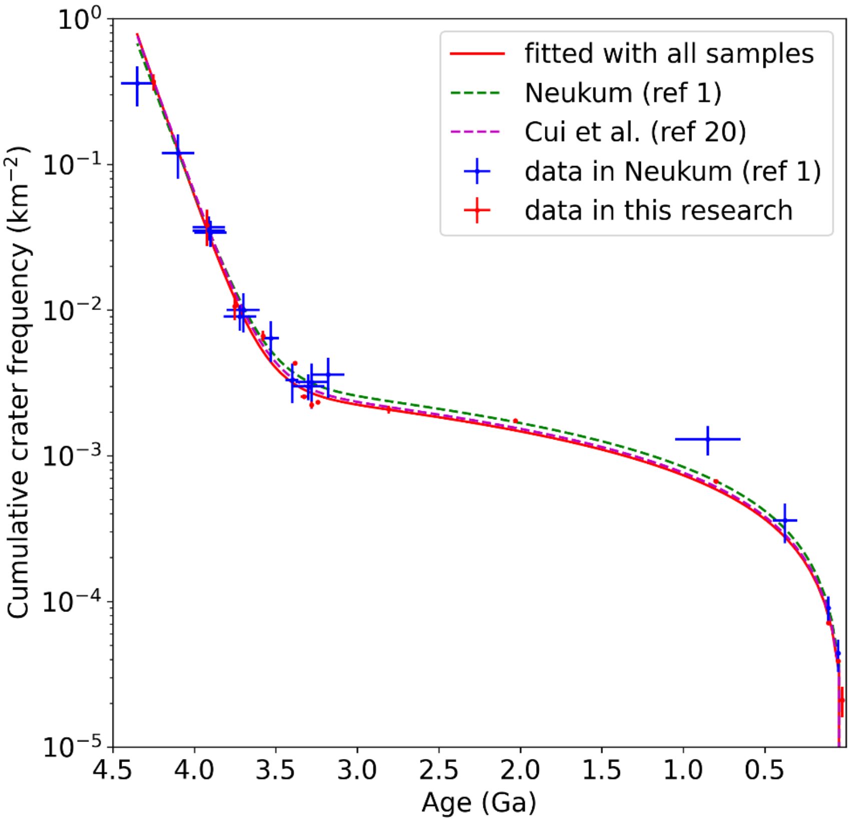

fig. S5. Comparison of the fitted lunar CF with all the lunar samples and the model by ref. (*20*), as well as the most widely used model by ref. (*1*). Note the model in ref. (*1*) is based only on the lunar nearside samples.

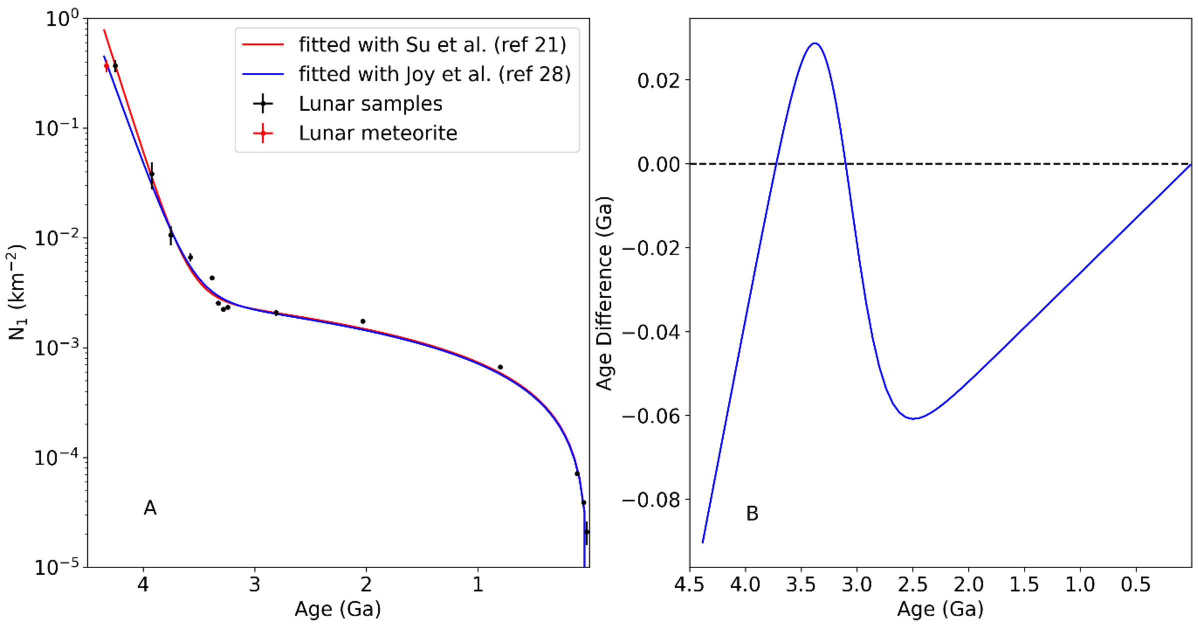

fig. S6. Comparison of refined models with CE-6 samples and lunar meteorite. (a) The refined lunar chronology model ($N(1,t) = 2.302 \times 10^{-13}(e^{6.504t} - 1) + 7.157 \times 10^{-4}t$) based on CE-6 samples(*21*) and lunar meteorite(*28*) along with all the radiometric ages. (b) The model age difference of the refined model with respect to the model based on ref. (*28*).

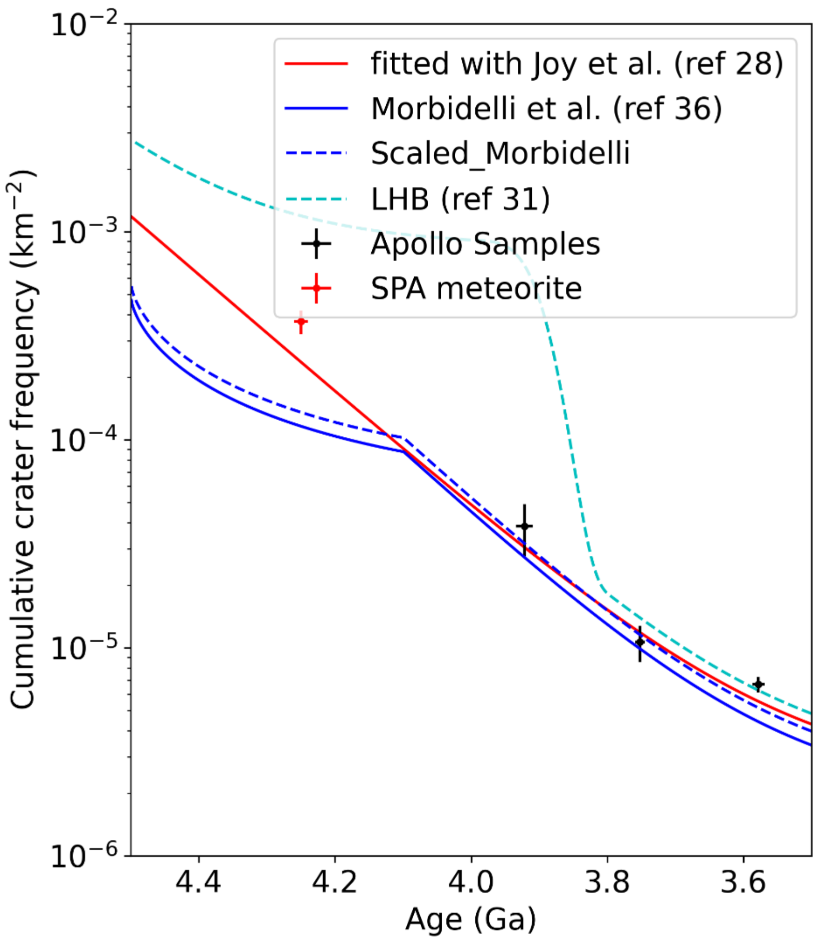

fig. S7. The comparison of the cumulative crater frequency *N*(>20 km) between previous models and the result based on the lunar samples except the SPA basin from lunar meteorite(*28*) in this study. The red solid line represents the result fitted with lunar samples and meteorite from ref. (*28*); the blue solid line is the result from ref. (*36*), and the blue dashed line represents the result normalized to this study based on the cumulative impact rate between 3.5 Ga and 4.1 Ga; the cyan dashed line shows the results for the Late Heavy Bombardment (LHB) from ref. (*31*). The black dots indicate measurements from Apollo samples, while the red cross represents the result from the lunar meteorite (*28*).

Supplementary code for the CF fitting

The code for the lunar CF fitting is shown in the 'RefinedCF.py'.

REFERENCES

1. G. Neukum, “Meteoriten bombardement und Datierung planetarer Oberflächen (Meteorite bombardment and dating of planetary surfaces),” thesis, Ludwig-Maximilians University, Munich, Germany (1983).

2. H. Hiesinger, J. W. Head III, U. Wolf, R. Jaumann, G. Neukum, Ages and stratigraphy of lunar mare basalts in Mare Frigoris and other nearside maria based on crater size-frequency distribution measurements. *J. Geophys. Res. Planets* **115**, E03003 (2010).

3. J. Whitten, J. W. Head, M. Staid, C. M. Pieters, J. Mustard, R. Clark, J. Nettles, R. L. Klima, L. Taylor, Lunar mare deposits associated with the Orientale impact basin: New insights into mineralogy, history, mode of emplacement, and relation to Orientale Basin evolution from Moon Mineralogy Mapper (M^3) data from Chandrayaan-1. *J. Geophys. Res. Planets* **116**, E00G09 (2011).

4. M. R. Kirchoff, C. R. Chapman, S. Marchi, K. M. Curtis, B. Enke, W. F. Bottke, Ages of large lunar impact craters and implications for bombardment during the Moon’s middle age. *Icarus* **225**, 325–341 (2013).

5. R. Wagner, J. W. Head III, U. Wolf, G. Neukum, Stratigraphic sequence and ages of volcanic units in the Gruithuisen region of the Moon. *J. Geophys. Res. Planets* **107**, 14-1–14-15 (2002).

6. M. Le Feuvre, M. A. Wieczorek, Nonuniform cratering of the Moon and a revised crater chronology of the inner Solar System. *Icarus* **214**, 1–20 (2011).

7. W. K. Hartmann, R. Strom, S. Weidenschilling, K. Blasius, K. Jones, in *Basaltic Volcanism on the Terrestrial Planets (Basaltic Volcanism Study Project)* (Pergamon Press, 1981), pp. 1050–1127.

8. D. Stöffler, G. Ryder, Stratigraphy and isotope ages of lunar geologic units: Chronological standard for the inner solar system. *Space Sci. Rev.* **96**, 9–54 (2001).

9. R. W. K. Potter, G. S. Collins, W. S. Kiefer, P. J. McGovern, D. A. Kring, Constraining the size of the South Pole-Aitken basin impact. *Icarus* **220**, 730–743 (2012).

10. R. I. Citron, D. E. Smith, S. T. Stewart, L. L. Hood, M. T. Zuber, The South Pole-Aitken basin: Constraints on impact excavation, melt, and ejecta. *Geophys. Res. Lett.* **51**, e2024GL110034 (2024).

11. W. M. Vaughan, J. W. Head, Impact melt differentiation in the South Pole-Aitken basin: Some observations and speculations. *Planet. Space Sci.* **91**, 101–106 (2014).

12. C. M. Pieters, J. W. Head, L. Gaddis, B. Jolliff, M. Duke, Rock types of South Pole-Aitken basin and extent of basaltic volcanism. *J. Geophys. Res. Planets* **106**, 28001–28022 (2001).

13. B. R. Hawke, C. A. Peterson, D. T. Blewett, D. B. J. Bussey, P. G. Lucey, G. J. Taylor, P. D. Spudis, Distribution and modes of occurrence of lunar anorthosite. *J. Geophys. Res. Planets* **108**, 5050 (2003).

14. J. H. Pasckert, H. Hiesinger, C. H. van der Bogert, Lunar farside volcanism in and around the South Pole–Aitken basin. *Icarus* **299**, 538–562 (2018).

15. X. Zeng, D. Liu, Y. Chen, Q. Zhou, X. Ren, Z. Zhang, W. Yan, W. Chen, Q. Wang, X. Deng, H. Hu, J. Liu, W. Zuo, J. W. Head, C. Li, Landing site of the Chang’e-6 lunar farside sample return mission from the Apollo basin. *Nat. Astron.* **7**, 1188–1197 (2023).

16. Y. Qian, J. Head, J. Michalski, X. Wang, C. H. van der Bogert, H. Hiesinger, L. Sun, W. Yang, L. Xiao, X. Li, G. Zhao, Long-lasting farside volcanism in the Apollo basin: Chang'e-6 landing site. *Earth Planet. Sci. Lett.* **637**, 118737 (2024).

17. Y. Wang, J. Nan, C. Zhao, B. Xie, S. Gou, Z. Yue, K. Di, H. Zhang, X. Deng, S. Sun, A catalogue of impact craters and surface age analysis in the Chang’e-6 landing area. *Remote Sens.* **16**, 2014 (2024).

18. C. Li, H. Hu, M.-F. Yang, J. Liu, Q. Zhou, X. Ren, B. Liu, D. Liu, X. Zeng, W. Zuo, G. Zhang, H. Zhang, S. Yang, Q. Wang, X. Deng, X. Gao, Y. Su, W. Wen, Z. Ouyang, Nature of the lunar farside samples returned by the Chang'E-6 mission. *Natl. Sci. Rev.* **11**, nwae328 (2024).

19. Q. W. L. Zhang, M.-H. Yang, Q.-L. Li, Y. Liu, Z.-Y. Yue, Q. Zhou, L.-Y. Chen, H.-X. Ma, S.-H. Yang, X. Tang, G.-L. Zhang, X. Ren, X.-H. Li, Lunar farside volcanism 2.8 billion years ago from Chang'e-6 basalts. *Nature* **643**, 356–360 (2025).

20. Z. Cui, Q. Yang, Y.-Q. Zhang, C. Wang, H. Xian, Z. Chen, Z. Xiao, Y. Qian, J. W. Head, C. R. Neal, L. Xiao, F. Luo, J. Chen, P. He, Y. Cao, Q. Zhou, F. Huang, L. Chen, B. Wei, J. Wang, Y.-N. Yang, S. Li, Y. Yang, X. Lin, J. Zhu, L. Zhang, Y.-G. Xu, A sample of the Moon's far side retrieved by Chang'e-6 contains 2.83-billion-year-old basalt. *Science* **386**, 1395–1399 (2024).

21. B. Su, Y. Chen, Z. Wang, D. Zhang, H. Chen, S. Gou, Z. Yue, Y. Liu, J. Yuan, G.-Q. Tang, S. Guo, Q. Li, Y.-T. Lin, X.-H. Li, F.-Y. Wu, South Pole–Aitken massive impact 4.25 billion years ago revealed by Chang'e-6. *Natl. Sci. Rev.* **12**, nwaf103 (2025).

22. S. Marchi, S. Mottola, G. Cremonese, M. Massironi, E. Martellato, A new chronology for the Moon and Mercury. *Astron. J.* **137**, 4936–4948 (2009).

23. S. J. Robbins, New crater calibrations for the lunar crater-age chronology. *Earth Planet. Sci. Lett.* **403**, 188–198 (2014).

24. H. Hiesinger, C. H. van der Bogert, J. H. Pasckert, L. Funcke, L. Giacomini, L. R. Ostrach, M. S. Robinson, How old are young lunar craters? *J. Geophys. Res. Planets* **117**, E00H10 (2012).

25. S. C. Werner, B. Bultel, T. Rolf, Review and revision of the lunar cratering chronology—Lunar timescale part 2. *Planet. Sci.* **4**, 147 (2023).

26. L. W. Bandermann, S. F. Singer, Calculation of meteoroid impacts on moon and earth. *Icarus* **19**, 108–113 (1973).

27. H. Li, N. Zhang, Z. Yue, Y. Zhang, Lunar cratering asymmetries with high lunar orbital obliquity and inclination of the Moon. *Res. Astron. Astrophys.* **21**, 140 (2021).

28. K. H. Joy, N. Wang, J. F. Snape, A. Goodwin, J. F. Pernet-Fisher, M. J. Whitehouse, Y. Liu, Y. T. Lin, J. R. Darling, P. Tar, R. Tartèse, Evidence of a 4.33 billion year age for the Moon's South Pole–Aitken basin. *Nat. Astron.* **9**, 55–65 (2024).

29. G. Neukum, B. A. Ivanov, W. K. Hartmann, Cratering records in the inner solar system in relation to the lunar reference system. *Space Sci. Rev.* **96**, 55–86 (2001).

30. W. K. Hartmann, Megaregolith evolution and cratering cataclysm models—Lunar cataclysm as a misconception (28 years later). *Meteorit. Planet. Sci.* **38**, 579–593 (2003).

31. G. Michael, A. Basilevsky, G. Neukum, On the history of the early meteoritic bombardment of the Moon: Was there a terminal lunar cataclysm? *Icarus* **302**, 80–103 (2018).

32. F. Tera, D. A. Papanastassiou, G. J. Wasserburg, Isotopic evidence for a terminal lunar cataclysm. *Earth Planet. Sci. Lett.* **22**, 1–21 (1974).

33. B. A. Cohen, T. D. Swindle, D. A. Kring, Support for the lunar cataclysm hypothesis from lunar meteorite impact melt ages. *Science* **290**, 1754–1756 (2000).

34. S. Marchi, W. F. Bottke, D. A. Kring, A. Morbidelli, The onset of the lunar cataclysm as recorded in its ancient crater populations. *Earth Planet. Sci. Lett.* **325-326**, 27–38 (2012).

35. M. D. Norman, A. A. Nemchin, A 4.2 billion year old impact basin on the Moon: U-Pb dating of zirconolite and apatite in lunar melt rock 67955. *Earth Planet. Sci. Lett.* **388**, 387–398 (2014).

36. A. Morbidelli, S. Marchi, W. F. Bottke, D. A. Kring, A sawtooth-like timeline for the first billion years of lunar bombardment. *Earth Planet. Sci. Lett.* **355-356**, 144–151 (2012).

37. B. A. Ivanov, Mars/Moon cratering rate ratio estimates. *Space Sci. Rev.* **96**, 87–104 (2001).

38. W. F. Bottke, D. Vokrouhlický, D. Minton, D. Nesvorný, A. Morbidelli, R. Brasser, B. Simonson, H. F. Levison, An Archaean heavy bombardment from a destabilized extension of the asteroid belt. *Nature* **485**, 78–81 (2012).

39. C. I. Fassett, D. A. Minton, Impact bombardment of the terrestrial planets and the early history of the Solar System. *Nat. Geosci.* **6**, 520–524 (2013).

40. T. F. Coleman, Y. Li, On the convergence of interior-reflective Newton methods for nonlinear minimization subject to bounds. *Math. Program.* **67**, 189–224 (1994).

41. T. F. Coleman, Y. Li, An interior trust region approach for nonlinear minimization subject to bounds. *SIAM J. Optim.* **6**, 418–445 (1996).

42. S. S. Shapiro, M. B. Wilk, An analysis of variance test for normality (complete samples). *Biometrika* **52**, 591–611 (1965).

43. A. Lagain, H. A. R. Devillepoix, P. Vernazza, D. Robertson, M. Granvik, P. Pokorny, A. Ozerov, P. M. Shober, L. Jorda, K. Servis, J. H. Fairweather, Y. Quesnel, G. K. Benedix, Recalibration of the lunar chronology due to spatial cratering-rate variability. *Icarus* **411**, 115956 (2024).

44. M. D. Norman, The lunar cataclysm: Reality or "mythconception"? *Elements* **5**, 23–28 (2009).

45. J. W. Head, Stratigraphy of the Descartes region (Apollo 16): Implications for the origin of samples. *The Moon* **11**, 77–99 (1974).

46. G. Neukum, B. A. Ivanov, in *Hazards Due to Comets and Asteroids*, T. Gehrels, M. S. Matthews, A. M. Schumann, Eds. (Univ. of Arizona Press, 1994), pp. 359–416.

47. D. Stöffler, A. Bischoff, R. Borchardt, A. Burghele, A. Deutsch, E. K. Jessberger, R. Ostertag, H. Palme, B. Spettel, W. U. Reimold, K. Wacker, H. Wänke, Composition and evolution of the lunar crust in the Descartes Highlands, Apollo 16. *J. Geophys. Res. Solid Earth* **90**, C449–C506 (1985).

48. D. E. Wilhelms, J. F. McCauley, N. J. Trask, *The Geologic History of the Moon* (US Government Printing Office, 1987).

49. G. Dalrymple, G. Ryder, R. Duncan, J. Huard, "^{40}Ar-^{39}Ar ages of Apollo 16 impact melt rocks by laser step heating," in the *32nd Lunar and Planetary Science Conference* (Lunar and Planetary Institute, 2001).

50. G. Ryder, Mass flux in the ancient Earth-Moon system and benign implications for the origin of life on Earth. *J. Geophys. Res. Planets* **107**, 6-1–6-13 (2002).

51. P. D. Spudis, Apollo 16 site geology and impact melts: Implications for the geologic history of the lunar highlands. *J. Geophys. Res. Solid Earth* **89**, C95–C107 (1984).

52. A. A. Nemchin, T. Long, B. L. Jolliff, Y. Wan, J. F. Snape, R. Zeigler, M. L. Grange, D. Liu, M. J. Whitehouse, N. E. Timms, F. Jourdan, Ages of lunar impact breccias: Limits for timing of the Imbrium impact. *Geochemistry* **81**, 125683 (2021).

53. A. A. Nemchin, R. T. Pidgeon, D. Healy, M. L. Grange, M. J. Whitehouse, J. Vaughan, The comparative behavior of apatite-zircon U-Pb systems in Apollo 14 breccias: Implications for the thermal history of the Fra Mauro Formation. *Meteorit. Planet. Sci.* **44**, 1717–1734 (2009).

54. R. E. Merle, A. A. Nemchin, M. L. Grange, M. J. Whitehouse, R. T. Pidgeon, High resolution U-Pb ages of Ca-phosphates in Apollo 14 breccias: Implications for the age of the Imbrium impact. *Meteorit. Planet. Sci.* **49**, 2241–2251 (2014).

55. S. C. Werner, B. Bultel, T. Rolf, V. Assis Fernandes, Orientale ejecta at the Apollo 14 landing site implies a 200-million-year stratigraphic time shift on the Moon. *Planet. Sci.* **3**, 65 (2022).

56. W. Iqbal, H. Hiesinger, D. Borisov, C. H. van der Bogert, J. W. Head, Geological mapping and chronology of lunar landing sites: Apollo 14. *Icarus* **406**, 115732 (2023).

57. J. M. Rhodes, N. J. Hubbard, H. Wiesmann, K. V. Rodgers, J. C. Brannon, B. M. Bansal, "Chemistry, classification, and petrogenesis of Apollo 17 mare basalts," in the *7th Lunar Science Conference* (Lunar and Planetary Institute/Pergamon Press, 1976).

58. W. M. Kaula, J. W. Head III, R. B. Merrill, R. O. Pepin, S. C. Solomon, D. Walker, C. A. Wood, *Basaltic Volcanism on the Terrestrial Planets* (Pergamon Press, 1981), 1286 pp.

59. J. F. Snape, A. A. Nemchin, M. J. Whitehouse, R. E. Merle, T. Hopkinson, M. Anand, The timing of basaltic volcanism at the Apollo landing sites. *Geochim. Cosmochim. Acta* **266**, 29–53 (2019).

60. B. Bultel, S. C. Werner, Sample-based spectral mapping around landing sites on the Moon—Lunar timescale part 1. *Planet. Sci. J.* **4**, 146 (2023).

61. W. Iqbal, H. Hiesinger, C. H. van der Bogert, Geological mapping and chronology of lunar landing sites: Apollo 11. *Icarus* **333**, 528–547 (2019).

62. H. H. Schmitt, N. E. Petro, R. A. Wells, M. S. Robinson, B. P. Weiss, C. M. Mercer, Revisiting the field geology of Taurus–Littrow. *Icarus* **298**, 2–33 (2017).

63. W. Iqbal, H. Hiesinger, C. van der Bogert, "New geological maps and crater size-frequency distribution measurements of the Apollo 17 landing site," in the *50th Lunar and Planetary Science Conference* (Lunar and Planetary Institute, 2019).

64. R. F. Dymek, A. L. Albee, A. A. Chodos, "Petrology and origin of Boulder #2 and #3, Apollo 17 Station 2," in the *7th Lunar Science Conference* (Lunar and Planetary Institute/Pergamon Press, 1976).

65. S. R. Winzer, D. F. Nava, P. J. Schuhmann, R. K. L. Lum, S. Schuhmann, M. M. Lindstrom, D. J. Lindstrom, J. A. Philpotts, The Apollo 17 "melt sheet": Chemistry, age and Rb/Sr systematics. *Earth Planet. Sci. Lett.* **33**, 389–400 (1977).

66. D. Stöffler, G. Ryder, B. A. Ivanov, N. A. Artemieva, M. J. Cintala, R. A. F. Grieve, Cratering history and lunar chronology. *Rev. Mineral. Geochem.* **60**, 519–596 (2006).

67. P. D. Spudis, G. Ryder, "Apollo 17 impact melts and their relation to the Serenitatis basin" in *Multi-ring basins: Formation and Evolution* (Pergamon Press, 1980).

68. P. D. Spudis, D. E. Wilhelms, M. S. Robinson, The Sculptured Hills of the Taurus Highlands: Implications for the relative age of Serenitatis, basin chronologies and the cratering history of the Moon. *J. Geophys. Res. Planets* **116**, E00H03 (2011).

69. C. M. Mercer, K. E. Young, J. R. Weirich, K. V. Hodges, B. L. Jolliff, J. A. Wartho, M. C. van Soest, Refining lunar impact chronology through high spatial resolution $^{40}Ar/^{39}Ar$ dating of impact melts. *Sci. Adv.* **1**, e1400050 (2015).

70. D. Beaty, A. Albee, "Comparative petrology of the Apollo 11 high-K basalts," in the *9th Lunar Science Conference* (Lunar and Planetary Institute, 1978).

71. E. A. Jerde, G. A. Snyder, L. A. Taylor, L. Yun-Gang, R. A. Schmitt, The origin and evolution of lunar high-Ti basalts: Periodic melting of a single source at Mare Tranquillitatis. *Geochim. Cosmochim. Acta* **58**, 515–527 (1994).

72. M. I. Staid, C. M. Pieters, J. W. Head III, Mare Tranquillitatis: Basalt emplacement history and relation to lunar samples. *J. Geophys. Res. Planets* **101**, 23213–23228 (1996).

73. G. Neukum, P. Horn, Effects of lava flows on lunar crater populations. *The Moon* **15**, 205–222 (1976).

74. J. Rhodes, J. Brannon, K. Rodgers, D. Blanchard, M. Dungan, "Chemistry of Apollo 12 mare basalts—Magma types and fractionation processes," in the *8th Lunar Science Conference* (Lunar and Planetary Institute, 1977).

75. L. E. Nyquist, J. L. Wooden, C. Y. Shih, H. Wiesmann, B. M. Bansal, Isotopic and REE studies of lunar basalt 12038: Implications for petrogenesis of aluminous mare basalts. *Earth Planet. Sci. Lett.* **55**, 335–355 (1981).

76. J. F. Snape, A. A. Nemchin, J. J. Bellucci, M. J. Whitehouse, R. Tartèse, J. J. Barnes, M. Anand, I. A. Crawford, K. H. Joy, Lunar basalt chronology, mantle differentiation and implications for determining the age of the Moon. *Earth Planet. Sci. Lett.* **451**, 149–158 (2016).

77. W. Iqbal, H. Hiesinger, C. H. van der Bogert, Geological mapping and chronology of lunar landing sites: Apollo 12. *Icarus* **352**, 113991 (2020).

78. A. P. Vinogradov, "Preliminary data on lunar ground brought to Earth by automatic probe," in the *2nd Lunar Science Conference* (Pergamon Press, 1971).

79. G. Kurat, A. Kracher, K. Keil, R. Warner, M. Prinz, "Composition and origin of Luna 16 aluminous mare basalts," in the *7th Lunar Science Conference* (Lunar and Planetary Institute/Pergamon Press, 1976).

80. J. C. Huneke, F. A. Podosek, G. J. Wasserburg, Gas retention and cosmic-ray exposure ages of a basalt fragment from Mare Fecunditatis. *Earth Planet. Sci. Lett.* **13**, 375–383 (1972).

81. D. A. Papanastassiou, G. J. Wasserburg, Rb-Sr age of a Luna 16 basalt and the model age of lunar soils. *Earth Planet. Sci. Lett.* **13**, 368–374 (1972).

82. B. A. Cohen, G. A. Snyder, C. M. Hall, L. A. Taylor, M. A. Nazarov, Argon-40-argon-39 chronology and petrogenesis along the eastern limb of the Moon from Luna 16, 20 and 24 samples. *Meteorit. Planet. Sci.* **36**, 1345–1366 (2001).

83. V. A. Fernandes, R. Burgess, Volcanism in Mare Fecunditatis and Mare Crisium: Ar-Ar age studies. *Geochim. Cosmochim. Acta* **69**, 4919–4934 (2005).

84. M. A. Ivanov, J. W. Head, H. Hiesinger, New insights into the regional and local geological context of the Luna 16 landing site. *Icarus* **400**, 115579 (2023).

85. Z. Yue, S. Sun, J. Du, S. Gou, K. Di, Y. Wang, Y. Lin, X. Li, F. Wu, New insights into the geological evolution history of Mare Fecunditatis. *Icarus* **425**, 116348 (2025).

86. L. S. Tarasov, M. A. Nazarov, I. D. Shevaleevskii, A. F. Kudriashova, A. S. Gaverdovskaia, M. I. Korina, "Mineralogy and petrography of lunar rocks from Mare Crisium (preliminary data)," in the *8th Lunar Science Conference* (Lunar and Planetary Institute, 1977).

87. A. L. Graham, R. V. Hutchison, Mineralogy and petrology of fragments From the Luna 24 core. *Philos. Trans. R. Soc. London Ser. A Math Phys. Eng. Sci.* **297**, 15–22 (1980).

88. A. Stettler, F. Albarède, ^{39}Ar-^{40}Ar systematics of two millimeter-sized rock fragments from Mare Crisium. *Earth Planet. Sci. Lett.* **38**, 401–406 (1978).

89. O. Schaeffer, A. Bence, J. Papike, D. Vaniman, "^{39}Ar-^{40}Ar and petrologic study of Luna 24 samples 24077,13 and 24077,63," in the *9th Lunar Science Conference* (Lunar and Planetary Institute, 1978).

90. R. Burgess, G. Turner, Laser argon-40-argon-39 age determinations of Luna 24 mare basalts. *Meteorit. Planet. Sci.* **33**, 921–935 (1998).

91. P. R. Renne, G. Balco, K. R. Ludwig, R. Mundil, K. Min, Response to the comment by W. H. Schwarz et al. on "Joint determination of ^{40}K decay constants and ^{40}Ar*/^{40}K for the Fish Canyon sanidine standard, and improved accuracy for ^{40}Ar/^{39}Ar geochronology" by P. R. Renne et al. (2010). *Geochim. Cosmochim. Acta* **75**, 5097–5100 (2011).

92. G. Neukum, B. König, J. Arkani-Hamed, A study of lunar impact crater size-distributions. *The Moon* **12**, 201–229 (1975).

93. F. K. Aitken, R. Brett, N. J. Hubbard, D. S. Mckay, C. P. Meyer, D. A. Morrison, E. Schonfeld, H. Takeda, "Mineralogy, chemistry, and origin of the KREEP component in soil samples from the Ocean of Storms," in the *2nd Lunar Science Conference* (Pergamon Press, 1971).

94. A. C. Stadermann, B. L. Jolliff, M. J. Krawczynski, C. W. Hamilton, J. J. Barnes, Analysis and experimental investigation of Apollo sample 12032,366-18, a chemically evolved basalt from the Moon. *Meteorit. Planet. Sci.* **57**, 794–816 (2022).

95. S. B. Simon, J. J. Papike, Petrology of the Apollo 12 highland component. *J. Geophys. Res. Solid Earth* **90**, 47–60 (1985).

96. P. Eberhardt, J. Geiss, N. Grögler, A. Stettler, How old is the crater copernicus? *The Moon* **8**, 104–114 (1973).

97. E. C. Alexander Jr., A. Bates, M. R. Coscio Jr., J. C. Dragon, V. R. Murthy, R. O. Pepin, T. R. Venkatesan, "K/Ar dating of lunar soils II," in the *7th Lunar Science Conference* (Lunar and Planetary Institute/Pergamon Press, 1976).

98. D. D. Bogard, D. H. Garrison, C. Y. Shih, L. E. Nyquist, ^{39}Ar-^{40}Ar dating of two lunar granites: The age of Copernicus. *Geochim. Cosmochim. Acta* **58**, 3093–3100 (1994).

99. G. Neukum, B. Koenig, "Dating of individual lunar craters," in the *7th Lunar Science Conference* (Lunar and Planetary Institute/Pergamon Press, 1976).

100. K. A. Howard, Avalanche mode of motion: Implications from lunar examples. *Science* **180**, 1052–1055 (1973).

101. R. Arvidson, R. Drozd, E. Guinness, C. Hohenberg, C. Morgan, R. Morrison, V. Oberbeck, "Cosmic ray exposure ages of Apollo 17 samples and the age of Tycho," in the *7th Lunar Science Conference* (Lunar and Planetary Institute/Pergamon Press, 1976).

102. B. K. Lucchitta, Crater clusters and light mantle at the Apollo 17 site; A result of secondary impact from Tycho. *Icarus* **30**, 80–96 (1977).

103. E. W. Wolfe, B. K. Lucchitta, V. S. Reed, G. E. Ulrich, A. G. Sanchez, "Geology of the Taurus-Littrow valley floor," in the *6th Lunar Science Conference* (Lunar and Planetary Institute, 1975).

104. R. J. Drozd, C. M. Hohenberg, C. J. Morgan, F. A. Podosek, M. L. Wroge, "Cosmic-ray exposure history at Taurus-Littrow," in the *8th Lunar Science Conference* (Lunar and Planetary Institute, 1977).

105. K. Marti, B. Lightner, T. Osborn, "Krypton and xenon in some lunar samples and the age of North Ray Crater," in the *4th Lunar Science Conference* (Pergamon Press, 1973).

106. C. Behrmann, G. Crozaz, R. Drozd, C. Hohenberg, C. Ralston, R. Walker, D. Yuhas, "Cosmic-ray exposure history of North Ray and South Ray material," in the *4th Lunar Science Conference* (Pergamon Press, 1973).

107. R. J. Drozd, C. M. Hohenberg, C. J. Morgan, C. Ralston, Cosmic-ray exposure history at the Apollo 16 and other lunar sites. Lunar surface dynamics. *Geochim. Cosmochim. Acta* **38**, 1625–1642 (1974).

108. R. Arvidson, G. Crozaz, R. J. Drozd, C. M. Hohenberg, C. J. Morgan, Cosmic ray exposure ages of features and events at the Apollo landing sites. *The Moon* **13**, 259–276 (1975).

109. H. J. Moore, J. M. Boyce, D. A. Hahn, Small impact craters in the lunar regolith—Their morphologies, relative ages, and rates of formation. *Moon Planets* **23**, 231–252 (1980).

110. R. A. F. Grieve, M. R. Dence, The terrestrial cratering record: II. The crater production rate. *Icarus* **38**, 230–242 (1979).

111. Y. Qian, L. Xiao, S. Yin, M. Zhang, S. Zhao, Y. Pang, J. Wang, G. Wang, J. W. Head, The regolith properties of the Chang'e-5 landing region and the ground drilling experiments using lunar regolith simulants. *Icarus* **337**, 113508 (2020).

112. Q.-L. Li, Q. Zhou, Y. Liu, Z. Xiao, Y. Lin, J.-H. Li, H.-X. Ma, G.-Q. Tang, S. Guo, X. Tang, J.-Y. Yuan, J. Li, F.-Y. Wu, Z. Ouyang, C. Li, X.-H. Li, Two-billion-year-old volcanism on the Moon from Chang'e-5 basalts. *Nature* **600**, 54–58 (2021).

113. X. Che, A. Nemchin, D. Liu, T. Long, C. Wang, M. D. Norman, K. H. Joy, R. Tartese, J. Head, B. Jolliff, J. F. Snape, C. R. Neal, M. J. Whitehouse, C. Crow, G. Benedix, F. Jourdan, Z. Yang, C. Yang, J. Liu, S. Xie, Z. Bao, R. Fan, D. Li, Z. Li, S. G. Webb, Age and composition of young basalts on the Moon, measured from samples returned by Chang'e-5. *Science* **374**, 887–890 (2021).

114. Z. Yue, K. Di, W. Wan, Z. Liu, S. Gou, B. Liu, M. Peng, Y. Wang, M. Jia, J. Liu, Z. Ouyang, Updated lunar cratering chronology model with the radiometric age of Chang'e-5 samples. *Nat. Astron.* **6**, 541–545 (2022).

115. M. Jia, Z. Yue, K. Di, B. Liu, J. Liu, G. Michael, A catalogue of impact craters larger than 200 m and surface age analysis in the Chang'e-5 landing area. *Earth Planet. Sci. Lett.* **541**, 116272 (2020).

116. Y. Qian, L. Xiao, S. Y. Zhao, J. N. Zhao, J. Huang, J. Flahaut, M. Martinot, J. W. Head, H. Hiesinger, G. X. Wang, Geology and scientific significance of the Rümker region in northern

Oceanus Procellarum: China's Chang'E-5 landing region. *J. Geophys. Res. Planets* **123**, 1407–1430 (2018).

117. B. Wu, J. Huang, Y. Li, Y. Wang, J. Peng, Rock abundance and crater density in the candidate Chang'E-5 landing region on the Moon. *J. Geophys. Res. Planets* **123**, 3256–3272 (2018).

118. Y. Qian, L. Xiao, J. W. Head, C. H. van der Bogert, H. Hiesinger, L. Wilson, Young lunar mare basalts in the Chang'e-5 sample return region, northern Oceanus Procellarum. *Earth Planet. Sci. Lett.* **555**, 116702 (2021).

119. F. Luo, Z. Xiao, Y. Wang, Y. Ma, R. Xu, S. Wang, M. Xie, Y. Wu, Q. Deng, P. Ma, The production population of impact craters in the Chang'E-6 landing mare. *Astrophys. J. Lett.* **974**, L37 (2024).

120. Z. Yue, S. Gou, S. Sun, W. Yang, Y. Chen, Y. Wang, H. Lin, K. Di, Y. Lin, X. Li, F. Wu, Geological context of the Chang'e-6 landing area and implications for sample analysis. *Int. J. Hydrogen Energ.* **5**, 100663 (2024).

121. I. Garrick-Bethell, K. Miljković, H. Hiesinger, C. H. van der Bogert, M. Laneuville, D. L. Shuster, D. G. Korycansky, Troctolite 76535: A sample of the Moon's South Pole-Aitken basin? *Icarus* **338**, 113430 (2020).

122. H. Hiesinger, C. van der Bogert, J. Pasckert, N. Schmedemann, M. Robinson, B. Jolliff, N. Petro, "New crater size-frequency distribution measurements of the South Pole-Aitken basin," in the *43rd Lunar and Planetary Science Conference* (Lunar and Planetary Institute, 2012).

123. C. I. Fassett, J. W. Head, S. J. Kadish, E. Mazarico, G. A. Neumann, D. E. Smith, M. T. Zuber, Lunar impact basins: Stratigraphy, sequence and ages from superposed impact crater populations measured from Lunar Orbiter Laser Altimeter (LOLA) data. *J. Geophys. Res. Planets* **117**, E00H06 (2012).

124. C. Orgel, G. Michael, C. I. Fassett, C. H. van der Bogert, C. Riedel, T. Kneissl, H. Hiesinger, Ancient bombardment of the inner solar system: Reinvestigation of the "fingerprints" of different impactor populations on the lunar surface. *J. Geophys. Res. Planets* **123**, 748–762 (2018).

125. T. Kneissl, G. G. Michael, N. Schmedemann, Treatment of non-sparse cratering in planetary surface dating. *Icarus* **277**, 187–195 (2016).

126. S. J. Robbins, A new global database of lunar impact craters >1–2 km: 1. Crater locations and sizes, comparisons with published databases, and global analysis. *J. Geophys. Res. Planets* **124**, 871–892 (2019).

127. R. V. Wagner, E. J. Speyerer, M. S. Robinson, the LROC Team, "New mosaicked data products from the LROC Team," in the *46th Lunar and Planetary Science Conference* (Lunar and Planetary Institute, 2015).

128. H. Sato, M. S. Robinson, S. J. Lawrence, B. W. Denevi, B. Hapke, B. L. Jolliff, H. Hiesinger, Lunar mare TiO_2 abundances estimated from UV/Vis reflectance. *Icarus* **296**, 216–238 (2017).
